# Toughening Self‐Healing Elastomers with Chain Mobility

**DOI:** 10.1002/advs.202308154

**Published:** 2024-06-12

**Authors:** Matthew Wei Ming Tan, Patrick Michael Thornton, Gurunathan Thangavel, Hyunwoo Bark, Reinhold Dauskardt, Pooi See Lee

**Affiliations:** ^1^ School of Materials Science and Engineering Nanyang Technological University 50 Nanyang Avenue Singapore 639798 Singapore; ^2^ Singapore‐HUJ Alliance for Research and Enterprise (SHARE), Smart Grippers for Soft Robotics (SGSR) Campus for Research Excellence and Technological Enterprise (CREATE) Singapore 138602 Singapore; ^3^ Department of Materials Science and Engineering Stanford University Stanford CA 94305 USA; ^4^ Present address: Advanced Materials Research Center Technology Innovation Institute (TII) Masdar City Abu Dhabi P.O Box 9639 United Arab Emirates

**Keywords:** chain mobility, elastomers, fracture toughness, hydrogen bonding, self‐healing

## Abstract

Enhancing fracture toughness and self‐healing within soft elastomers is crucial to prolonging the operational lifetimes of soft devices. Herein, it is revealed that tuning the polymer chain mobilities of carboxylated‐functionalized polyurethane through incorporating plasticizers or thermal treatment can enhance these properties. Self‐healing is promoted as polymer chains gain greater mobility toward the broken interface to reassociate their bonds. Raising the temperature from 80 to 120 °C, the recovered work of fracture is increased from 2.86 to 123.7 MJ m^−3^. Improved fracture toughness is realized through two effects. First, strong carboxyl hydrogen bonds dissipate large energies when broken. Second, chain mobilities enable the redistribution of localized stress concentrations to allow crack blunting, enlarging the size of dissipation zones. At optimal conditions of plasticizers (3 wt.%) or temperature (40 °C) to promote chain mobilities, fracture toughness improves from 16.3 to 19.9 and 25.6 kJ m^−2^, respectively. Insights of fracture properties at healed soft interfaces are revealed through double cantilever beam tests. These measurements indicate that fracture mechanics play a critical role in delaying complete failure at partial self‐healing. By imparting optimal polymer chain mobilities within tough and self‐healing elastomers, effective prevention against damage and better recovery are realized.

## Graphical abstract

This study demonstrates that tuning the polymer chain mobilities of carboxylated‐functionalized polyurethane can improve both self‐healing abilities and fracture toughness. Higher mobilities facilitate enhanced reassociation of bonds at the damaged interface and enable redistribution of localized stress concentrations, increasing crack blunting. Healed soft interfaces are investigated, emphasizing the impact of fracture mechanics in delaying complete failure at partial self‐healing.

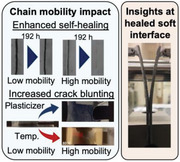

## 1 Introduction

Strong and tough elastomers are critical for soft devices to achieve higher durability in applications such as soft robotics,^[^ ^1^, ^2^ ^]^ wearable electronics,^[^ ^3^, ^4^ ^]^ and biomedical devices.^[^ ^5^, ^6^ ^]^ In addition, imparting self‐healing capabilities can drastically improve the lifetime of these devices, restoring their function after failure.^[^ ^4^, ^7^ ^]^ However, enhancing these properties simultaneously within a single material remains challenging. Improved fracture toughness and strength can be achieved by incorporating reinforcing phases from stiff fibers to nanosheets within a soft matrix.^[^ ^8^, ^9^ ^]^ Alternatively, double networks have been adopted, in which a brittle, covalent, crosslinked first network dissipates large energies when ruptured. In contrast, the second network, which is soft and ductile, maintains the structural integrity of the matrix.^[^ ^10^, ^11^ ^]^ When covalent bonds are ruptured, the material is permanently damaged and unable to recover its properties. To exhibit self‐healing, elastomers are required to possess reversible bonds and sufficient polymer chain mobility to enable their reassociation at the broken interface.^[^ ^12^ ^]^ As a result, a conflict between self‐healing and high mechanical properties arises as strong reversible bonds tend to limit the mobility of polymer chains.^[^ ^13^, ^14^ ^]^ To address this, external stimuli such as temperature,^[^ ^15^ ^]^ humidity,^[^ ^16^ ^]^ and solvents^[^ ^17^ ^]^ have been utilized to promote chain mobility for healing. Recent efforts have also focused on tuning the intrinsic structures of polymers to tackle this conflict, including designing microphase‐separated structures, combining multiple dynamic bonds, and incorporating dangling chains.^[^ ^14^, ^18^, ^19^ ^]^ To achieve high fracture toughness and self‐healing capabilities, supramolecular interactions such as hydrogen bonds and metal–ligand coordination are introduced. These interactions can be disrupted to dissipate energy at the crack tip to impart high fracture toughness and able to reform to restore their properties.^[^ ^4^, ^20^ ^]^ However, few studies correlate these two properties, especially at the healed interface. The utilization of broadband dielectric spectroscopy has provided valuable insights at the healed region, revealing disparities in the polymer network structure between pristine and healed samples.^[^ ^21^, ^22^ ^]^ From a practical aspect, the alignment of broken elastomers is often imperfect, and misalignments may introduce artificial defects where failure begins.^[^ ^23^ ^]^ This emphasizes on the need for further investigations, particularly aimed at understanding and preventing failure at the weakly healed interface.

Microphase‐separated structures composed of hard and soft domains are increasingly adopted by elastomers to concurrently realize favorable tensile strength, fracture toughness, and self‐healing capabilities, owing to their highly tunable nature.^[^ ^24^, ^25^, ^26^ ^]^ These hard domains generally act as physical crosslinks that provide reinforcing effects, while the soft domains form the matrix and enable stretchability. Dispersed hard domains have been described to impart notch insensitivity through crack‐bridging effects that transfer stress concentrations at the crack tip to the surrounding elastomer network.^[^ ^24^ ^]^ Also, Zhuo et al. revealed that stiffness mismatches between soft and hard domains promoted crack deflection and branching for enhanced fracture toughness, similar to stiff fibers toughening a soft matrix.^[^ ^27^ ^]^ Soft segments are further found to impart strain‐hardening behaviors, owing to crystallization due to the alignment of polymer chains along the stretching direction.^[^ ^28^, ^29^ ^]^ To facilitate energy dissipation and chain mobilities, supramolecular interactions of different strengths have been incorporated into hard domains.^[^ ^4^, ^28^, ^30^, ^31^ ^]^ For instance, cooperative effects from strong quadruple and weak hydrogen bond motifs provided elastomers with improved strength and promoted fast self‐healing, respectively.^[^ ^25^, ^31^ ^]^ These multi‐strength hydrogen bonds allowed energy dissipation over small and large deformations, enhancing tear resistance. However, these approaches often require complicated synthesis procedures that may hinder large‐scale production.

Herein, we introduce an approach to elevate the self‐healing capabilities and fracture toughness of these microphase‐separated elastomers through promoting polymer chain mobilities. The impact of chain mobility on carboxyl‐functionalized polyurethane (CPU) is evaluated by introducing polyethylene glycol (PEG) plasticizers or raising the temperatures during measurements. CPU possesses an intrinsic microphase‐separated structure at which strong carboxyl and weaker urethane hydrogen bonds are incorporated into hard domains to impart high strength, work of fracture, and self‐healing properties to the elastomer.^[^ ^32^ ^]^ Self‐healing capabilities are enhanced with increasing chain mobilities, as polymer chains can easily diffuse toward the broken interface for bond reassociation. The rupture of strong hydrogen bonds formed between carboxyl functionalities from cyclic tensile tests showed large energy dissipation, a critical component often incorporated to achieving high fracture toughness. However, despite the addition of low amounts of PEG plasticizers (3 wt.%) that led to the lowering of energy dissipation, fracture toughness increased from 16.3 to 19.9 kJ m^−2^. The impact of chain mobilities was further validated with pure shear tests of CPU at differing temperatures, at which fracture toughness of 25.6 kJ m^−2^ was achieved at 40 °C. These findings can be attributed to increased crack blunting that distributes large local stress concentrations. As such, this work highlights the importance of chain mobilities to achieve strong, crack‐resistant, and self‐healable elastomers, extending current design principles that emphasize large energy dissipation and strong reversible bonds. Notably, while most works focus on tensile tests to understand the self‐healing properties, this work provides deeper insights into the crack propagation at healed interfaces through correlations with double cantilever beam (DCB) tests of self‐adhered CPU. We reveal that the fracture toughness of the elastomer plays a critical role in delaying the complete failure of the elastomer, especially at low degrees of healing. With this insight, future soft devices can potentially be imparted with improved fracture toughness and self‐healing capabilities through optimal control of polymer chain mobilities.

## 2 Results and Discussion

### 2.1 Characterization of CPU and CPU‐PEG Blends

**Figure 1:**
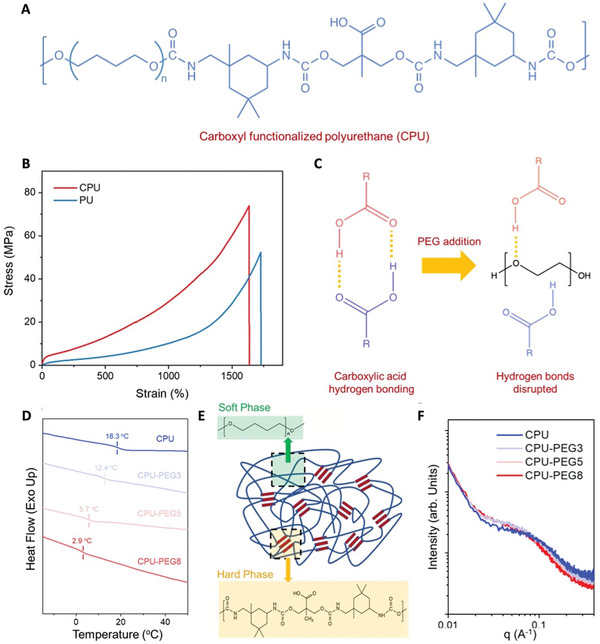
Molecular structure and material characterization. A) Chemical structure of CPU. B) Tensile stress–strain curves of CPU and control PU. C) Schematic illustration of PEG plasticizer disrupting the hydrogen bonds formed between carboxyl functional groups. D) DSC curves of CPU and CPU‐PEG blends (3, 5, and 8 wt.%) with at heating rate of 10 °C min^−1^ from −15 to 60 °C. E) Schematic of micro‐phase separated CPU with hard and soft phases. F) SAXS profile of CPU and CPU‐PEG blends (3, 5, and 8 wt.%).

CPU was synthesized through a facile one‐pot reaction between polytetrahydrofuran (PTMG, average M_n_ ≈ 1000 Da), 2,2‐bis(hydroxymethyl)propionic acid (DMPA), isophorone diisocyanate (IPDI) and catalyst dibutyltin dilaurate (DBDTL) (**Figure** **1**A). Confirmation of the successful synthesis of CPU was obtained through ^1^H nuclear magnetic resonance and Fourier transform infrared spectroscopy measurements (Figures S1 and S2, Supporting Information). Control polyurethanes (PU) were prepared through a similar synthetic route, except that DMPA was replaced with 2‐methyl‐1,3‐propanediol (Figure S3, Supporting Information). Based on the tensile tests (Figure 1B), CPU displayed a larger tensile strength (72.6 MPa) and work of fracture (433.9 MJ m^−3^) compared to PU due to hydrogen bonds provided by carboxyl groups that acted as strong physical crosslinks. CPU and PU showed similar molecular weights and polydispersity index (Table S1, Supporting Information), further supporting that the enhanced mechanical properties are due to carboxyl groups rather than potential entanglement effects. After establishing the high mechanical performance of CPU, the role of chain mobilities on fracture toughness and self‐healing capabilities was investigated. The properties may be tuned by varying the content of carboxyl groups, at which lower carboxyl groups lead to lower tensile strengths (Figure S4, Supporting Information). However, this requires the synthesis of multiple batches of CPUs with different carboxyl content, making preparation tedious and impractical. Instead, an efficient and generic strategy to promote chain mobilities is to introduce PEG plasticizers to a single batch of CPU, forming polymer blends of CPU‐PEGX, with X referring to the concentration of PEG. The plasticizing effects of PEG were attributed to its ability to disrupt carboxyl hydrogen bonds between CPU chains (Figure 1C).^[^ ^33^, ^34^ ^]^ Glass transition temperatures (T_g_) were also reduced from 18.3 to 2.9 °C with increasing PEG content, as measured from differential scanning calorimetry (DSC) (Figure 1D), as lower energy was required for molecular motion with PEG. The disruption of carboxyl hydrogen bond is supported by the FTIR spectra peak deconvolutions. At C═O regions (1800–1600 cm^−1^), peak shifts to higher frequencies occurred, suggesting a weakening of hydrogen bonding between carboxyl groups (Figure S5, Supporting Information).^[^ ^32^, ^35^ ^]^ The properties of these polyurethanes are closely related to their microphase‐separated structure of hard and soft domains (Figure 1E), which is facilitated by the hydrogen bonding between hard segments. These structures were verified with small‐angle X‐ray scattering (SAXS) studies that revealed broad scattering peaks in all CPU‐PEG films (Figure 1F). In addition, small peak shifts toward lower *q* with PEG addition indicate that the average distance between hard microdomains was lengthened.^[^ ^36^ ^]^ Thermogravimetric analysis further displayed a two‐stage degradation corresponding to hard and soft segments (Figure S6, Supporting Information).^[^ ^37^ ^]^ CPU‐PEG films were highly transparent, with an average transmittance above 90% at the visible light wavelength (400–750 nm) (Figure S7, Supporting Information).

### 2.2 Mechanical and Self‐Healing Properties

**Figure 2:**
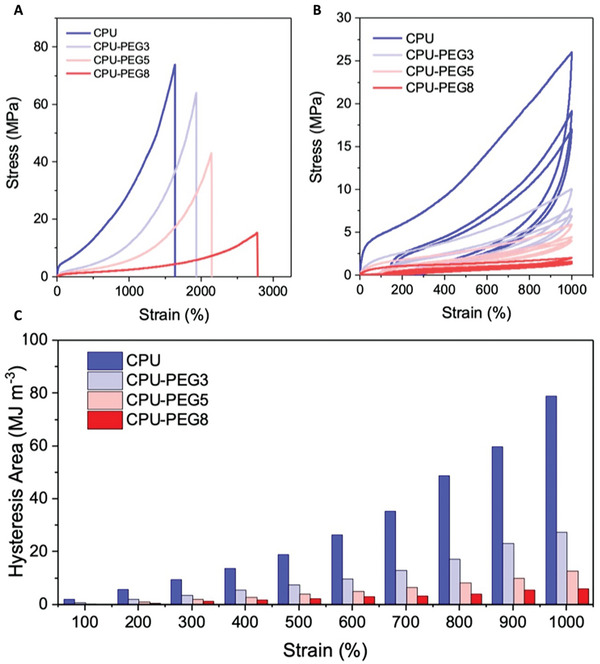
Mechanical properties of CPU and CPU‐PEG blends. A) Representative tensile stress–strain curves at a strain rate of 100 mm min^−1^. B) Beginning three cycles of cyclic tensile tests with a strain limit of 1000%. C) Hysteresis areas of the first cycle of cyclic tensile tests at various limiting strains between 100 to 1000%.

As revealed from the tensile tests (**Figure** **2**A), the mechanical properties of CPU‐PEG blends depended closely on the amount of carboxyl hydrogen bonds that formed physical crosslinks. Increasing the PEG plasticizers disrupted a larger extent of carboxyl hydrogen bonds,^[^ ^33^ ^]^ leading to tensile strength reductions from 72.6 to 15.9 MPa. However, the stretchability increased from 1676% to 2788% due to greater sliding between polymer chains. Considering these two properties, the work of fracture based on integrating the stress–strain curve was reduced from 433.9 to 132.6 MJ m^−3^ at higher PEG concentrations (Figure S8, Supporting Information). The mechanical properties of the elastomers are shown in Table S2 (Supporting Information). Cyclic tensile tests were performed to investigate the energy dissipation behaviors of the elastomers. All samples displayed a large hysteresis area during the first loading due to the energy dissipated from the rupture of hydrogen bonds (Figure 2B; Figure S9, Supporting Information). When the second loading was conducted immediately after, a significant decline in the hysteresis was observed as these bonds could not be reconstructed in time.^[^ ^38^, ^39^ ^]^ However, after a short rest period of 1 h, stress–strain behaviors were completely recovered for all samples based on the overlapping hysteresis areas and negligible residual strains (Figures S10 and S11, Supporting Information). This recoverability of CPU‐PEG elastomers can be associated with the dynamic nature of hydrogen bonds that reformed after unloading.^[^ ^15^, ^38^ ^]^ In addition, aliphatic hexatomic IPDI provides higher flexibility within hard segments to restore their bonds.^[^ ^39^ ^]^ Measured at various limiting strains, hysteresis areas of CPU increased gradually to 400% before rapidly increasing at higher strains (Figure 2C; Figure S12, Supporting Information). Hysteresis areas at low strains were mainly associated with the rupture of weak urethane hydrogen bonds. In contrast, energy dissipation contributions at increased strains resulted from carboxyl hydrogen bond rupture.^[^ ^15^, ^17^ ^]^ This was further supported by the lowered hysteresis areas at all strain limits by introducing PEG plasticizers that minimized carboxyl hydrogen bonds and lowered internal frictions between polymer chains.

**Figure 3:**
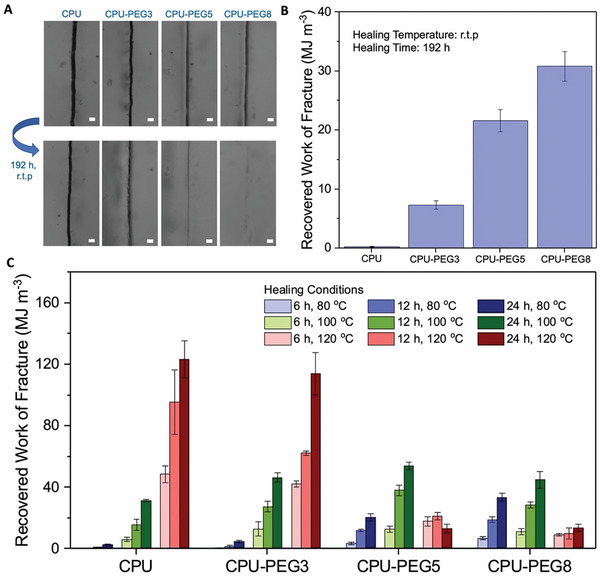
Self‐healing of CPU and CPU‐PEG blends. A) Optical image of damaged films (top) and after self‐healing (bottom) for 192 h at room temperature (r.t.p). Scale bar represents 10 µm. B) Recovered work to fracture after healing dumbbell‐shaped samples for 192 h at room temperature (r.t.p). C) Recovered work to fracture after healing dumbbell‐shaped samples for various durations (6, 12, and 24 h) at elevated temperatures (80, 100, and 120 °C). All error bars are the standard deviation of three independent samples.

The ability of hydrogen bonds to reassociate themselves imparts the elastomers with self‐healing capabilities. At room temperature, the CPU showed minimal signs of healing even after 192 h, as optical micrographs at the surface of the damaged interface showed no change (**Figure** **3**A). By increasing PEG concentration, this interface gradually faded away to form a faint scar, indicating improved healing capabilities. To determine the recovery of the mechanical performance, dumbbell‐shaped films were cut into half, rejoined, and healed under various conditions before performing tensile tests (Figure 3B; Figure S13, Supporting Information). CPU showed negligible healing at room temperature due to the lack of chain mobility from the large amounts of strong physical crosslinks, hindering the reassociation of bonds at the damage site.^[^ ^12^, ^40^ ^]^ On the other hand, chain mobilities can be activated with PEG plasticizers to enable self‐healing capabilities. The recovered work of fracture based on the integration of stress–strain curves of healed samples after 192 h, was increased to 7.4, 21.7, and 30.9 MJ m^−3^ for CPU‐PEG3, CPU‐PEG5, and CPU‐PEG8 respectively. These findings relate to investigations into the molecular dynamic of polymers, revealing slow chain segment motions due to restricted networks from crosslinks.^[^ ^21^, ^41^ ^]^ To promote chain mobilities for greater recovery of mechanical properties, higher healing temperatures (80, 100, and 120 °C) were applied for different durations (Figure 3C). At 80 °C, CPU exhibited self‐healing capabilities, and all samples achieved greater recovered work of fracture, with CPU‐PEG8 displaying the highest recovered work of fracture (33.7 MJ m^−3^) within 24 h (Figure S14, Supporting Information). As temperatures were raised to 100 and 120 °C, the highest recovered work of fracture shifted to CPU‐PEG5 (54.2 MJ m^−3^) and CPU (123.7 MJ m^−3^), respectively (Figures S15 and S16, Supporting Information). This can be attributed to increased chain mobility at higher temperatures, easing the restoration of their strong initial mechanical properties (even with low content or no PEG). In addition, the mechanical properties of CPU‐PEG5 and CPU‐PEG8 degraded significantly after being subjected to 120 °C. This may be due to the high content of plasticizers that hindered the reformation of carboxyl hydrogen bonds after being thermally dissociated. Nonetheless, with improved chain mobilities from plasticizers or elevated temperatures, the self‐healing capabilities of the elastomers can be significantly enhanced. The healing efficiencies of the elastomers are summarized in Table S3 (Supporting Information). Comparing with recent polymers possessing self‐healing capabilities, the elastomers in this work display a favorable combination of high tensile strength and work of fracture, preventing damage from first occurring (Figure S17, Supporting Information). This is balanced with the capacity to recover notable mechanical properties after self‐healing at elevated temperatures, extending its utility post‐damage.

### 2.3 Fracture Toughness

**Figure 4:**
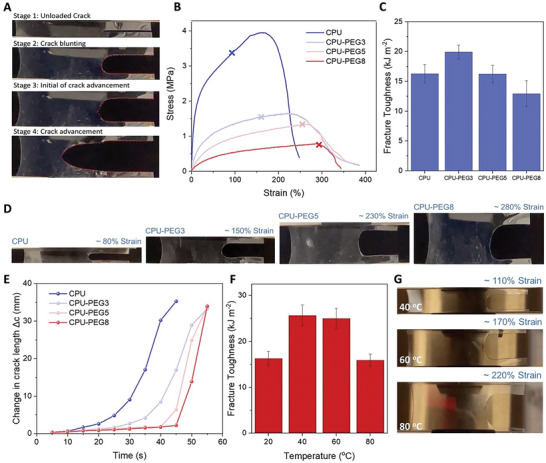
Fracture toughness behaviors of CPU and CPU‐PEG blends. A) Stages of crack evolution in CPU‐PEG elastomers. B) Stress–strain curves of notched samples under pure shear test. The cross indicates the point where crack advancement was initiated, and the corresponding strain represents the critical strain. C) Fracture toughness of CPU and CPU‐PEG blends (3, 5, and 8 wt.%). D) Digital images of notched CPU and CPU‐PEG samples at maximum strains at which crack blunting occurs. E) Change in crack length of notched CPU and CPU‐PEG films against time. F) Fracture toughness of CPU at various temperatures (40, 60, and 80 °C). G) Digital images of notched CPU samples at the maximum strains at which crack blunting occurs when subjected to various temperatures (40, 60, and 80 °C). All error bars are the standard deviation of three independent samples.

The fracture toughness of the elastomers was evaluated through a pure shear test devised by Rivlin and Thomas, to ensure minimal dependence of the energy release rate on the crack length.^[^ ^42^, ^43^ ^]^ The crack evolution in CPU‐PEG elastomers can be generally categorized into four stages (**Figure** **4**A; Movie S1, Supporting Information).^[^ ^44^ ^]^ Starting from an unloaded crack (stage 1), initial loading led to blunting of the crack (stage 2), forming a semi‐circular shape with minimal advancement. Upon subsequent loading, a sharp notch began to form within the semi‐circular‐shaped crack tip (stage 3). At this stage, the strain energy at the damaged region is sufficient to create a stress concentration that turns into a running crack at increased loading (stage 4). Pure shear tests were performed on notched samples to identify the critical strain occurring at stage 3 (Figure 4B). To determine the fracture toughness of the elastomers, the work of fracture was calculated based on integrating the stress–strain curves of unnotched films up to the critical strain (Figure S18, Supporting Information).^[^ ^44^, ^45^ ^]^ As such, CPU displayed a large fracture toughness of 16.3 kJ m^−2^ due to strong carboxyl hydrogen bonds that dissipate large amounts of energy when ruptured (Figure 4C).^[^ ^4^, ^15^, ^39^ ^]^ This is supported from cyclic tensile tests that show the highest hysteresis among the elastomers tested. Despite this, notched CPU displayed minimal crack blunting and early crack advancement at low strains. In contrast, CPU‐PEG blends showed increasing crack blunting with PEG concentration (Figure 4D), at which crack propagation occurred only at higher strains. This is attributed to the improved chain mobility that allowed homogenous dispersion of stress concentrations at the crack tip, forming a larger energy dissipative zone that shielded the crack from remote loading (Figure S19, Supporting Information).^[^ ^43^, ^46^ ^]^ Thus, increased energy dissipation from strong carboxyl groups and enlarged dissipation zone size from chain mobility led to fracture toughness enhancement of CPU‐PEG3 to 19.9 kJ m^−2^. As the PEG was further increased (5 and 8 wt.%), the disruptive effect of plasticizers on carboxyl hydrogen bonding dominated, causing a deterioration in the work of fracture and, thus, the fracture toughness. Crack propagation behaviors through the width were evaluated by monitoring the crack length evolution with time (Figure 4E). While CPU displayed early crack advancement (15 s), greater resistance toward crack propagation was achieved from the strength of hard domains that resist and block the crack growth throughout its path. This is similarly exhibited by tough elastomers that contain hydrogen bond‐rich domains that hinder crack growth, closely emulating a soft matrix reinforced with hard components.^[^ ^47^, ^48^ ^]^ As PEG concentrations increased to 3, 5, and 8 wt.%, more hard domains were disrupted across the width, causing crack propagation to occur faster (30, 15, and 10 s, respectively). Therefore, through optimum control over the chain mobility of such microphase‐separated structures, resistance to crack propagation can be realized.

Pure shear tests were performed on CPU at elevated temperatures from 40 to 80 °C to further verify the impact of polymer chain mobility on fracture toughness. At elevated temperatures, the hydrogen bond equilibrium shifts toward the dissociated state, resulting in greater chain mobility and reduced mechanical properties.^[^ ^2^, ^49^ ^]^ Based on pure shear tests (Figure S20, Supporting Information), the fracture toughness of CPU increased to 25.6 kJ m^−2^ at 40 °C before declining to 25.0 and 15.9 kJ m^−2^ at 60 and 80 °C, respectively (Figure 4F). Analogous to increased plasticizer concentrations, a greater extent of crack blunting within CPU was observed at higher temperatures (Figure 4G). As a result, stress concentration was alleviated at the crack tip to allow higher critical strains to be realized for improved fracture toughness. Also, the crack propagation time was reduced with temperatures as minimal hard domains were present to block the crack growth (Figure S21, Supporting Information). These findings prove the importance of providing sufficient chain mobilities within microphase‐separated elastomers to improve fracture properties.

### 2.4 Fracture Toughness of Healed Interfaces

**Figure 5:**
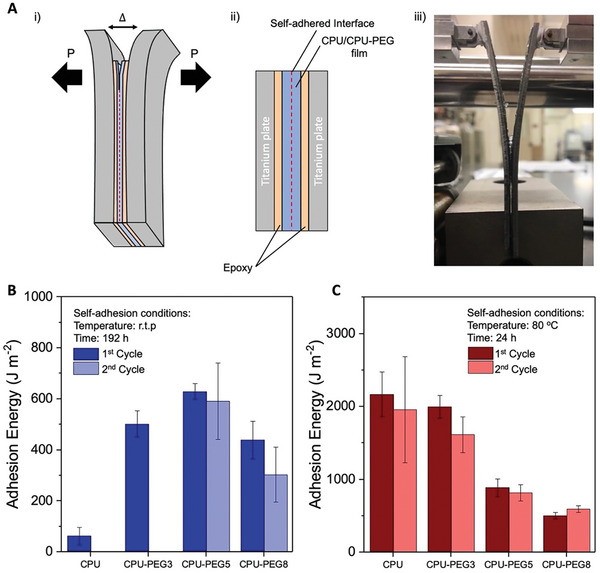
Double cantilever beam (DCB) measurements of CPU and CPU‐PEG blends. A) i) Schematic of DCB specimen, with *P* and *Δ* representing the load and displacement. ii) Schematic of DCB specimen cross‐section. iii) Digital image of DCB measurements. B) Adhesion energy of CPU and CPU‐PEG blends (3, 5, and 8 wt.%) for two self‐adhesion cycles for 192 h at room temperature (r.t.p). No adhesion was achieved for the second self‐adhesion cycle for CPU and CPU‐PEG3 due to the low chain mobility. C) Adhesion energy of CPU and CPU‐PEG blends (3, 5, and 8 wt.%) for two self‐adhesion cycles for 24 h at 80 °C.

Practically, healed interfaces often display misalignments that introduce artificial defects where cracks may be initiated, leading to lower healing efficiencies.^[^ ^23^, ^50^, ^51^ ^]^ To provide insights into the fracture mechanics of healed interfaces, DCB tests were performed on self‐adhered elastomers (**Figure** **5**A). While self‐adhesion and self‐healing of elastomers differ in terms of the starting number of unassociated hydrogen bonds and their equilibrium states,^[^ ^52^ ^]^ the role of chain mobility in promoting hydrogen bond formation across interfaces are similar.^[^ ^53^ ^]^ As such, adhesion energies derived from self‐adhered elastomers in DCB tests provide insights into the fracture toughness of healed interfaces. This DCB configuration is a well‐established procedure to characterize the adhesion fracture energy of interfaces at a controlled rate.^[^ ^54^, ^55^ ^]^ Through adopting DCB configurations to soft stretchable elastomers, toughening effects arising from chain alignments or modulus changes occurring with strain can be decoupled.^[^ ^56^ ^]^ In addition, defects from misalignment of healed interfaces can be minimized due to the large contact areas. These factors enable greater accuracy in understanding the fracture toughness of healed interfaces. Elastomer films were bonded to two separate titanium substrates before being placed in firm contact for healing at various conditions. A notch was created at the self‐adhered interface and a uniaxial load was applied perpendicularly to the elastomer films, driving crack propagation in a controlled manner. DCB measurements were repeated for a second cycle to determine the recovery performance of the adhered interface. The adhesion energies are related to both the energy to break the associated bonds across the adhered interface and to create new surfaces, along with the energy dissipation zone surrounding the crack tip.^[^ ^54^ ^]^ When DCB samples were adhered at room temperature for 192 h (Figure 5B), CPU showed the lowest adhesion energy (<100 J m^−2^) due to limited chain mobilities that prevented bond association. Unlike self‐healing tensile tests, the highest adhesion energy was achieved by CPU‐PEG5, as shown in Figure 5B, instead of CPU‐PEG8, which had the highest chain mobilities. At room temperature with a lower degree of bond association, the total adhesion energy will be highly dependent on both the total energy required to break the associated bonds at the interface and the energy dissipation zone at the crack tip. As the PEG concentration increased, the chain mobility was enhanced, increasing the number of associated bonds, and expanding the energy dissipation zone. However, the intrinsic bond strength decreases through the disruption of hydrogen bonds, reducing the overall energy required to rupture these associated bonds. This implies that an optimal amount of PEG plasticizers was required to maximize the total adhesion energy when healing is minimal at room temperatures. When adhered at 80 °C for 24 h, CPU and CPU‐PEG3 achieved high adhesion energies of 2170 and 2000 J m^−2^, respectively, with a small reduction after the second cycle (Figure 5C). These adhesion energy trends are closely similar to that of pure shear tests, indicating that failure at interfaces with high degree of bond association primarily depends on the energy dissipation behaviors at the crack tip. However, the size of energy dissipation zones was suppressed due to mechanical constraints imposed by the DCB configuration.^[^ ^57^, ^58^ ^]^ This is evident at higher PEG concentrations (5 and 8 wt.%), at which adhesion energies rapidly declined to 890 and 505 J m^−2^, respectively. In contrast, strong dynamic bonds that dissipate large energies when ruptured, such as in CPU, may be better suited for these constraint conditions. Through DCB studies, it can be correlated that under partial self‐healing, improved fracture toughness aids in delaying premature failures at the interface. In contrast, at complete self‐healing, failure conditions follow the bulk properties.

## 3 Conclusion

By promoting polymer chain mobilities, self‐healing and fracture toughness can be enhanced. In addition, high strength and work to fracture were attained through introducing carboxyl functional groups within hard domains of polyurethane. The improvements to self‐healing can be associated with easier mobility of polymer chains to the broken interface to reassociate its bonds. On the other hand, enhanced fracture toughness was attributed to two factors. First, the strong carboxyl hydrogen bonds dissipated large amounts of energy at the crack tip when ruptured. Second, the enhanced chain mobilities allowed local stresses to be homogenously distributed, expanding the dissipation zone and increasing crack blunting. As a result, the crack tip was effectively shielded from driving forces supplied by remote loads. Furthermore, DCB measurements were introduced for the first time to correlate the fracture toughness of self‐adhered interface of soft stretchable elastomers to the healed interface of tensile test samples. At room temperatures where healing effects were lower, an optimal amount of PEG plasticizers was required to maximize the energy needed to separate the healed bonds and the energy dissipation zone surrounding the crack tip. In contrast, thermally healed interfaces showed trends similar to the fracture behaviors of the pristine specimens. This is attributed to the successfully reformed carboxyl hydrogen bonds that enabled significant energy dissipation when ruptured, contributing to high adhesion energies. DCB measurements highlight the importance of evaluating the healed interfaces, often the critical source of weakness in self‐healed elastomers. Future works may explore lowering the temperature requirements to induce self‐healing, autonomous crack closure, and alignment of damaged parts, as well as the impact of chain mobilities on fatigue resistance. By imparting enhanced chain mobilities to micro‐phase separated elastomers, a pathway to improved elastomers with damage prevention and recovery can be realized, potentially enhancing application for soft robots and wearables electronics.

## 4 Experimental Section

### Synthesis of CPU and CPU‐PEG Blends

PTMG (24 mmol) was vacuum dried for 1 h at 110 °C under Ar atmosphere to remove moisture residues. Next, DMF (24 ml), IPDI (72 mmol), DMPA (24 mmol), and DBTDL (10 µl) were added and stirred for 24 h at 80 °C under argon atmosphere. To quench the remaining isocyanate groups, methanol was added. A viscous layer formed and settled to the bottom, at which the top solution was decanted. The remaining methanol was removed through vacuum drying and DMF was added to redissolve the product. To form CPU‐PEG blends, differing amount of PEG (3, 5, and 8 wt.%) was added to CPU and mixed. CPU‐PEG blends were blade‐coated onto a glass plate and placed in an oven at 80 °C for 12 h to obtain a thin film.

### Mechanical and Self‐Healing Measurements

Dumbbell‐shaped samples for tensile tests were prepared based on ASTM D638‐14, Type V. Static mechanical tester (MTS Criterion Model 43, MTS Systems Corporation) was used to obtain stress–strain curves at a strain rate of 100 mm min^−1^. The work of fracture was achieved by integrating the stress–strain curve up to the strain at break. Cyclic tensile test measurements were performed by stretching the samples to a specified limiting strain at the same strain rate. The hysteresis area was obtained by subtracting the area under the loading curve by the area under the unloading curve. Self‐healing tests were conducted by cutting dumbbell‐shaped samples into two separate pieces using a sharp razor blade. This ensures that the broken interface can be flat and smooth to provide maximum surface contact for healing to take place. After which, gentle pressure was immediately applied, to manually to bring the two parts into contact, allowing self‐healing to occur at various times and temperatures. For temperature‐induced self‐healing, samples were placed in an oven after the two broken parts were placed into contact. The healed samples were tested under the same conditions as tensile tests to determine the recovered mechanical performance and based on three independent samples.

### Fracture Toughness Measurements

To determine the fracture toughness of the elastomers, pure shear tests were performed at a strain rate of 20 mm min^−1^ with a static tensile tester (MTS Criterion Model 43, MTS Systems Corporation). Unnotched samples were prepared with a width and thickness of 60 mm and ≈200 µm. The initial distance between the grippers (H_0_) was maintained at 5 mm. Notched samples were prepared based on the same dimensions with a crack length of 20 mm. The crack evolution behavior of notched samples was recorded with a video camera at 60 frames per second. The critical strain (λ_c_) was identified as the strain at which the crack begins to advance. Subsequently, the strain energy density (W(λ_c_)) was determined by the integral of unnotched samples up to the critical strain. As derived by Rivlin and Thomas, the fracture toughness (G) can be obtained from Equation 1.

(1) $$G\;=\;W\left(\lambda_{c}\right)H_{0}$$

To perform pure shear tests at elevated temperatures, an environmental chamber accessory was used with the static tensile tester. Before each measurement, samples were kept at the desired testing temperature (40, 60, or 80 °C) for at least 15 min to ensure that a steady state was reached. Fracture toughness measurements are based on three independent samples.

### Double Cantilever Beam (DCB) Measurements

The samples for DCB measurements were prepared by adhering the elastomer films onto two rectangular titanium substrates using a two‐part epoxy (DP8005‐black, 3 m). Because of the large adhesion energy differences, a 1.6 mm thick beam was utilized for the CPU and CPU‐PEG3 samples whereas a 0.8 mm thick beam was used for the CPU‐PEG5 and CPU‐PEG8 samples to maximize deflection while remaining in the elastic regime. Subsequently, the two films were placed in slightly weighted contact and left to self‐adhere under various conditions. Tests were conducted at a constant rate of 10 µm s^−1^. The DCB methodology for measuring adhesion energies is based on linear elastic fracture mechanics.^[^ ^59^, ^60^ ^]^ Multiple measurement cycles, of at least three cycles, were conducted in a given sample at various crack lengths as the crack propagated, and the average and standard deviation of the calculated adhesion energies constitute the reported value and error bars, respectively. Only the second healing of CPU‐PEG5 at 80 °C was based on the average of two cycles due to sample failure at the epoxy. Adhesion energies (G_A_) were derived from Equation 2 where *P_c_* and *a* refer to the critical load and de‐bond length at crack propagation, respectively. Also, *b*, *h*, and *E’* represent the width, thickness, and plane strain Young modulus of the titanium substrate, respectively.

(2) $$G_{A}=\;\frac{12P_{C}^{2}a^{2}}{b^{2}h^{3}E^{\prime }}{\left(1+\frac{0.64h}{a}\right)}^{2}$$

## Conflict of Interest

The authors declare no conflict of interest.

## Author Contributions

M.W.M.T., R.D., and P.S.L. performed conceptualization; M.W.M.T. and P.M.T. performed methodology; M.W.M.T., P.M.T., G.T., and H.B. performed investigation; M.W.M.T. wrote the original draft; M.W.M.T., P.M.T., R.D., and P.S.L. wrote the original draft and reviewed and edited the final manuscript; R.D. and P.S.L. did supervision; P.S.L. acquired funding.

## Supporting information

Supporting Information

Supplemental Movie 1

## Data Availability

The data that support the findings of this study are available from the corresponding author upon reasonable request.

## References

[1] M. T. Tolley , R. F. Shepherd , B. Mosadegh , K. C. Galloway , M. Wehner , M. Karpelson , R. J. Wood , G. M. Whitesides , Soft Rob. 2014, 1, 213.

[2] M. W. M. Tan , H. Bark , G. Thangavel , X. Gong , P. S. Lee , Nat. Commun. 2022, 13, 6769. 36351948 10.1038/s41467-022-34301-w PMC9646827

[3] C. L. He , F. C. Liang , L. Veeramuthu , C. J. Cho , J. S. Benas , Y. R. Tzeng , Y. L. Tseng , W. C. Chen , A. Rwei , C. C. Kuo , Adv. Sci. 2021, 8, 2102275. 10.1002/advs.202102275 PMC8564429 34519441

[4] J. Kang , D. Son , G. J. N. Wang , Y. Liu , J. Lopez , Y. Kim , J. Y. Oh , T. Katsumata , J. Mun , Y. Lee , Adv. Mater. 2018, 30, 1706846. 10.1002/adma.201706846 29424026

[5] D. Zhalmuratova , H.‐J. Chung , ACS Appl. Polym. Mater. 2020, 2, 1073.

[6] Y. Wang , G. A. Ameer , B. J. Sheppard , R. Langer , Nat. Biotechnol. 2002, 20, 602. 12042865 10.1038/nbt0602-602

[7] S. Terryn , J. Brancart , D. Lefeber , G. Van Assche , B. Vanderborght , Sci. Rob. 2017, 2, eaan4268. 10.1126/scirobotics.aan4268 33157852

[8] Z. Wang , C. Xiang , X. Yao , P. L. Floch , J. Mendez , Z. Suo , Proc. Natl. Acad. Sci. USA 2019, 116, 5967. 30850517 10.1073/pnas.1821420116 PMC6442615

[9] J. Peng , A. P. Tomsia , L. Jiang , B. Z. Tang , Q. Cheng , Nat. Commun. 2021, 12, 1. 34315892 10.1038/s41467-021-24835-w PMC8316440

[10] D. R. King , T. Okumura , R. Takahashi , T. Kurokawa , J. P. Gong , ACS Appl. Mater. Interfaces 2019, 11, 35343. 31475822 10.1021/acsami.9b12935

[11] J. P. Gong , Science 2014, 344, 161.24723604

[12] A. Campanella , D. Döhler , W. H. Binder , Macromol. Rapid Commun. 2018, 39, 1700739. 10.1002/marc.201700739 29337415

[13] C. Kim , N. Yoshie , Polym. J. 2018, 50, 919.

[14] A. M. Wemyss , C. Bowen , C. Plesse , C. Vancaeyzeele , G. T. Nguyen , F. Vidal , C. Wan , Mater. Sci. Engin.: R: Rep. 2020, 141, 100561.

[15] Y. Song , Y. Liu , T. Qi , G. L. Li , Angew. Chem., Int. Ed. 2018, 57, 13838. 10.1002/anie.201807622 30144244

[16] J. Xu , S. Ye , J. Fu , J. Mater. Chem. A 2018, 6, 24291.

[17] M. W. M. Tan , G. Thangavel , P. S. Lee , Adv. Funct. Mater. 2021, 31, 2103097.

[18] S. Utrera‐Barrios , R. Verdejo , M. A. López‐Manchado , M. H. Santana , Mater. Horiz. 2020, 7, 2882. 10.1039/d3mh01312j 37997164

[19] Z.‐W. An , R. Xue , K. Ye , H. Zhao , Y. Liu , P. Li , Z.‐M. Chen , C.‐X. Huang , G.‐H. Hu , Nanoscale 2023, 15, 6505. 36883369 10.1039/d2nr07110j

[20] C. Xing , H. Wu , R. Du , Q. Zhang , X. Jia , Polym. Chem. 2021, 12, 4778.

[21] M. Hernández , A. Grande , S. van der Zwaag , S. J. Garcia , ACS Appl. Mater. Interfaces 2016, 8, 10647. 27057588 10.1021/acsami.6b02259

[22] M. A. Sattar , S. Gangadharan , A. Patnaik , ACS Omega 2019, 4, 10939. 31460192 10.1021/acsomega.9b01243 PMC6648382

[23] R. H. Aguirresarobe , S. Nevejans , B. Reck , L. Irusta , H. Sardon , J. M. Asua , N. Ballard , Prog. Polym. Sci. 2021, 114, 101362.

[24] D. Wang , J. Xu , J. Chen , P. Hu , Y. Wang , W. Jiang , J. Fu , Adv. Funct. Mater. 2020, 30, 1907109.

[25] K. Xu , G. Chen , M. Zhao , W. He , Q. Hu , Y. Pu , RSC Adv. 2022, 12, 2712. 35425297 10.1039/d1ra07083e PMC8979244

[26] Y. Lai , X. Kuang , P. Zhu , M. Huang , X. Dong , D. Wang , Adv. Mater. 2018, 30, 1802556. 10.1002/adma.201802556 30073707

[27] Y. Zhuo , Z. Xia , Y. Qi , T. Sumigawa , J. Wu , P. Šesták , Y. Lu , V. Håkonsen , T. Li , F. Wang , Adv. Mater. 2021, 33, 2008523. 10.1002/adma.202008523 PMC11468028 33938044

[28] T. Yang , X. Lu , X. Wang , Y. Li , X. Wei , W. Wang , J. Sun , ACS Appl. Polym. Mater. 2023, 5, 2830.

[29] Y. Li , W. Li , A. Sun , M. Jing , X. Liu , L. Wei , K. Wu , Q. Fu , Mater. Horiz. 2021, 8, 267. 34821305 10.1039/d0mh01447h

[30] Y. Huang , H. Wu , W. Li , Z. Yuan , Q. Wu , R. Li , J. Wu , J. Mater. Chem. A 2022, 10, 24290.

[31] X. Yan , Z. Liu , Q. Zhang , J. Lopez , H. Wang , H.‐C. Wu , S. Niu , H. Yan , S. Wang , T. Lei , J. Am. Chem. Soc. 2018, 140, 5280. 29595956 10.1021/jacs.8b01682

[32] S. Park , G. Thangavel , K. Parida , S. Li , P. S. Lee , Adv. Mater. 2019, 31, 1805536. 10.1002/adma.201805536 30387213

[33] M. Tian , B. Yan , Y. Yao , L. Zhang , T. Nishi , N. Ning , J. Mater. Chem. C 2014, 2, 8388.

[34] M. G. A. Vieira , M. A. da Silva , L. O. dos Santos , M. M. Beppu , Eur. Polym. J. 2011, 47, 254.

[35] D. Chattopadhyay , A. J. Muehlberg , D. C. Webster , Prog. Org. Coat. 2008, 63, 405.

[36] N. Hossieny , V. Shaayegan , A. Ameli , M. Saniei , C. Park , Polymer 2017, 112, 208.

[37] M. Sáenz‐Pérez , J. M. Laza , J. García‐Barrasa , J. L. Vilas , L. M. Leon , Polym. Eng. Sci. 2018, 58, 238.

[38] M.‐C. Luo , J. Zeng , Z.‐T. Xie , L.‐Y. Wei , G. Huang , J. Wu , Polymer 2016, 105, 221.

[39] Z. Li , Y. L. Zhu , W. Niu , X. Yang , Z. Jiang , Z. Y. Lu , X. Liu , J. Sun , Adv. Mater. 2021, 33, 2101498. 10.1002/adma.202101498 34062022

[40] Y. Pan , J. Hu , Z. Yang , L. Tan , ACS Appl. Polym. Mater. 2019, 1, 425.

[41] S. Utrera‐Barrios , R. Verdugo Manzanares , J. Araujo‐Morera , S. González , R. Verdejo , M. Á. López‐Manchado , M. Hernández Santana , Polymers 2021, 13, 3234. 34641050 10.3390/polym13193234 PMC8512226

[42] R. Rivlin , A. G. Thomas , J. Polym. Sci. 1953, 10, 291.

[43] R. Long , C.‐Y. Hui , Soft Matter 2016, 12, 8069. 27714361 10.1039/c6sm01694d

[44] F. Luo , T. L. Sun , T. Nakajima , T. Kurokawa , Y. Zhao , A. B. Ihsan , H. L. Guo , X. F. Li , J. P. Gong , Macromolecules 2014, 47, 6037.

[45] J.‐Y. Sun , X. Zhao , W. R. Illeperuma , O. Chaudhuri , K. H. Oh , D. J. Mooney , J. J. Vlassak , Z. Suo , Nature 2012, 489, 133. 22955625 10.1038/nature11409 PMC3642868

[46] W. Li , S. Zheng , X. Zou , Y. Ren , Z. Liu , W. Peng , X. Wang , D. Liu , Z. Shen , Y. Hu , Adv. Funct. Mater. 2022, 32, 2207348.

[47] H. Chen , J. J. Koh , M. Liu , P. Li , X. Fan , S. Liu , J. C. Yeo , Y. Tan , B. C. Tee , C. He , ACS Appl. Mater. Interfaces 2020, 12, 31975. 32536151 10.1021/acsami.0c08213

[48] R. Guo , Q. Zhang , Y. Wu , H. Chen , Y. Liu , J. Wang , X. Duan , Q. Chen , Z. Ge , Y. Zhang , Adv. Mater. 2023, 2212130. 10.1002/adma.202212130 36822221

[49] F. Song , Z. Li , P. Jia , M. Zhang , C. Bo , G. Feng , L. Hu , Y. Zhou , J. Mater. Chem. A 2019, 7, 13400.

[50] L. F. Fan , M. Z. Rong , M. Q. Zhang , X. D. Chen , ACS Appl. Mater. Interfaces 2018, 10, 38538. 30284805 10.1021/acsami.8b15636

[51] X. Xun , Z. Zhang , X. Zhao , B. Zhao , F. Gao , Z. Kang , Q. Liao , Y. Zhang , ACS Nano 2020, 14, 9066. 32658455 10.1021/acsnano.0c04158

[52] E. B. Stukalin , L.‐H. Cai , N. A. Kumar , L. Leibler , M. Rubinstein , Macromolecules 2013, 46, 7525. 10.1021/ma401111n PMC3857756 24347684

[53] A. Faghihnejad , K. E. Feldman , J. Yu , M. V. Tirrell , J. N. Israelachvili , C. J. Hawker , E. J. Kramer , H. Zeng , Adv. Funct. Mater. 2014, 24, 2322.

[54] S. R. Dupont , M. Oliver , F. C. Krebs , R. H. Dauskardt , Sol. Energy Mater. Sol. Cells 2012, 97, 171.

[55] K. Lionti , L. Cui , W. Volksen , R. Dauskardt , G. Dubois , B. Toury , ACS Appl. Mater. Interfaces 2013, 5, 11276. 24090249 10.1021/am403506k

[56] A. Klein , P. G. Whitten , K. Resch , G. Pinter , J. Polym. Sci., Part B: Polym. Phys. 2015, 53, 1763.

[57] P. Y. Yuen , R. H. Dauskardt , Macromol. Mater. Eng. 2016, 301, 1096.

[58] P. Y. Yuen , S. L. Moffitt , F. D. Novoa , L. T. Schelhas , R. H. Dauskardt , Prog. Photovoltaics 2019, 27, 693.

[59] M. Kanninen , Int. J. Fract. 1973, 9, 83.

[60] T. L. Anderson , Fracture Mechanics: Fundamentals And Applications, CRC Press, Boca Raton, FL 2017.

