# Supplementary material for: Toughening Self‐Healing Elastomers with Chain Mobility
Source: Adv Sci (Weinh). 2024 Jun 12;11(30):2308154. doi: 10.1002/advs.202308154 (PMC11321685; doi:10.1002/advs.202308154)
Supplement: Supplementary file 1 — Supporting Information [file ADVS-11-2308154-s001.pdf]

ADVANCED SCIENCE
Open Access

# Supporting Information

for *Adv. Sci.*, DOI 10.1002/advs.202308154

Toughening Self-Healing Elastomers with Chain Mobility

*Matthew Wei Ming Tan, Patrick Michael Thornton, Gurunathan Thangavel, Hyunwoo Bark, Reinhold Dauskardt* and Pooi See Lee**

Supporting Information

# Toughening Self-healing Elastomers with Chain Mobility

Matthew Wei Ming Tan,[1,2] Patrick Michael Thornton,[3] Gurunathan Thangavel,[1]‡ Hyunwoo Bark,[1] Reinhold Dauskardt,[3]* Pooi See Lee[1,2]*

[1]School of Materials Science and Engineering, Nanyang Technological University, 50 Nanyang Avenue, Singapore 639798, Singapore

[2]Singapore-HUJ Alliance for Research and Enterprise (SHARE), Smart Grippers for Soft Robotics (SGSR), Campus for Research Excellence and Technological Enterprise (CREATE), Singapore, 138602, Singapore

[3]Department of Materials Science and Engineering, Stanford University, Stanford, CA 94305, USA.

*Corresponding author: Reinhold Dauskardt, rhd@stanford.edu; Pooi See Lee, pslee@ntu.edu.sg

‡ Present address: Advanced Materials Research Center, Technology Innovation Institute (TII), Masdar City, Abu Dhabi, P.O Box 9639, United Arab Emirates

## Materials

Polytetrahydrofuran glycol (PTMG, average Mn ~ 1000 Da), isophorone diisocyanate (IPDI, 98%, mixture of isomers), 2,2-bis(hydroxymethyl)propionic acid (DMPA), 2-methyl-1,3-propanediol, dibutyltin dilaurate (DBTDL), and dimethylformamide (DMF, anhydrous 99.8%) were purchased from Sigma Aldrich. Polyethylene glycol (PEG, average Mn ~ 1000 Da) was purchased from Fluka. Deuterated solvents for NMR analysis were obtained from Cambridge Isotope Laboratories, Inc.

## Synthesis of control polyurethane

PTMG (24 mmol) was vacuum dried for 1 h at 110 $^{o}$C under Ar atmosphere to remove moisture residues. Next, DMF (24 ml), IPDI (72 mmol), 2-methyl-1,3-propanediol (24 mmol) and DBTDL (10 ml) were added and stirred for 24 h at 80 $^{o}$C under argon atmosphere. To quench the remaining isocyanate groups, methanol was added. A viscous layer formed and settled to the bottom, at which the top solution was decanted. The remaining methanol was removed through vacuum drying and DMF was added to dissolve the product. Control polyurethane was then blade-coated onto a glass plate and placed in an oven at 80 $^{o}$C for 12 h to obtain a thin film.

## Material Characterization

$^{1}$H nuclear magnetic resonance spectrometers (NMR, 400 MHz Bruker DPX 400) and FTIR (FTIR-ATR, Perkin Elmer, Frontier) were utilized to investigate the chemical linkages of the synthesized CPU and CPU-PEG blends. $^{1}$H-NMR were carried out at room temperature using the deuterated solvents and tetramethylsilane (TMS) as the internal standard. Thermal transitions were measured through dynamic scanning calorimetry (DSC) using TA Instruments DSC Q10. DSC measurements were performed at a heating rate of 10 $^{o}$C min$^{-1}$ under a nitrogen atmosphere. Microphase separation of the CPU and CPU-PEG blends were analyzed through small angle X-ray scattering (SAXS) measurements using SWAXS Xenocs Nanoinxider. Thermogravimetric analysis (TGA) was performed using TA Instruments Q50 at a heating rate of 10 $^{o}$C min$^{-1}$ from 40 to 550 $^{o}$C. UV-Vis spectrum was obtained from UV-Vis NIR Spectrophotometer (Lambda950) from 250 to 800 nm. Molecular weights were determined using gel permeation chromatography (GPC, Agilent 1260). GPC of all samples were run using chloroform as the mobile phase with a flow rate of 1 mL min$^{-1}$. Monodispersed polystyrenes were used for the calibration curves.

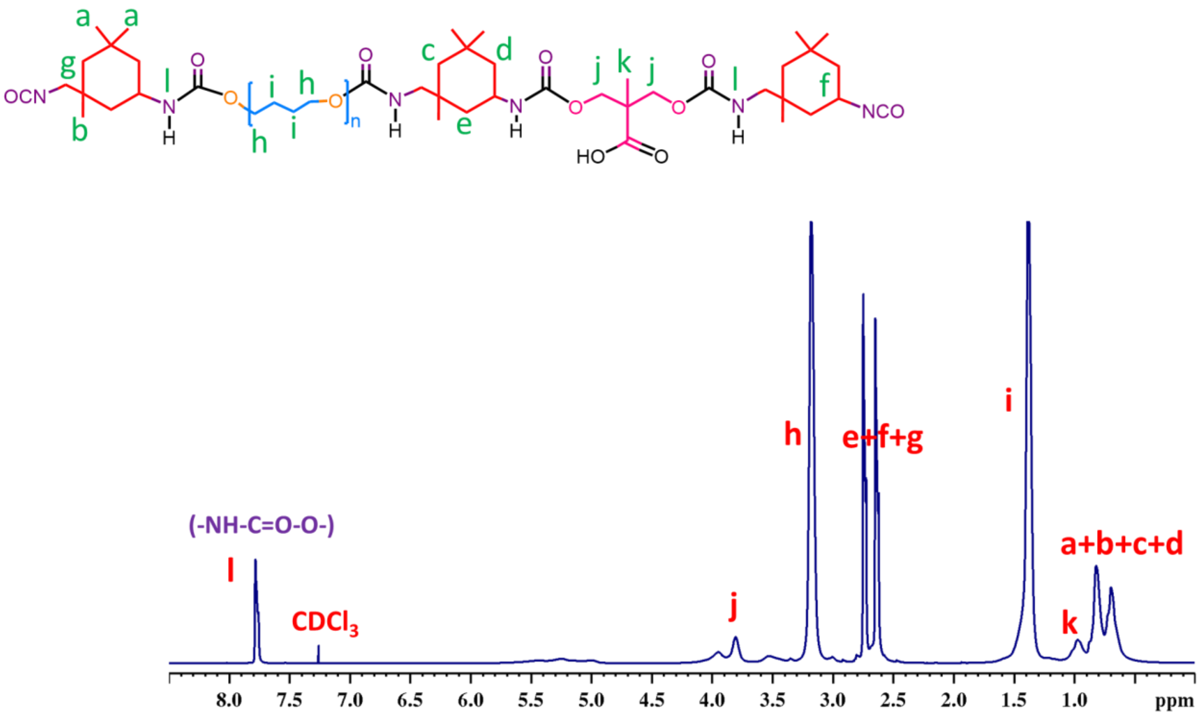


**Figure S1.** $^{1}$H-NMR spectra of carboxyl-functionalized polyurethane (CPU) dispersed in deuterated $CDCl_3$. $^{1}$H NMR of CPU (400 MHz, CDCl3, 25 $^{o}$C) δ (ppm): 7.81 (s, 1H, -NH-C=O-O-), 3.92 (s, 2H, -CH2-O-), 3.33 (s, 4H, -CH2-CH2-O-), 2.83 (m, 2H, -CH2-NCO), 2.71 (d, 2H, C-CH2-C), 1.45 (m, 4H, -CH2-CH2-CH2), 1.02 (s, 3H, CH3-C-), 0.67-1.01 (s, 9H, -C-CH3).

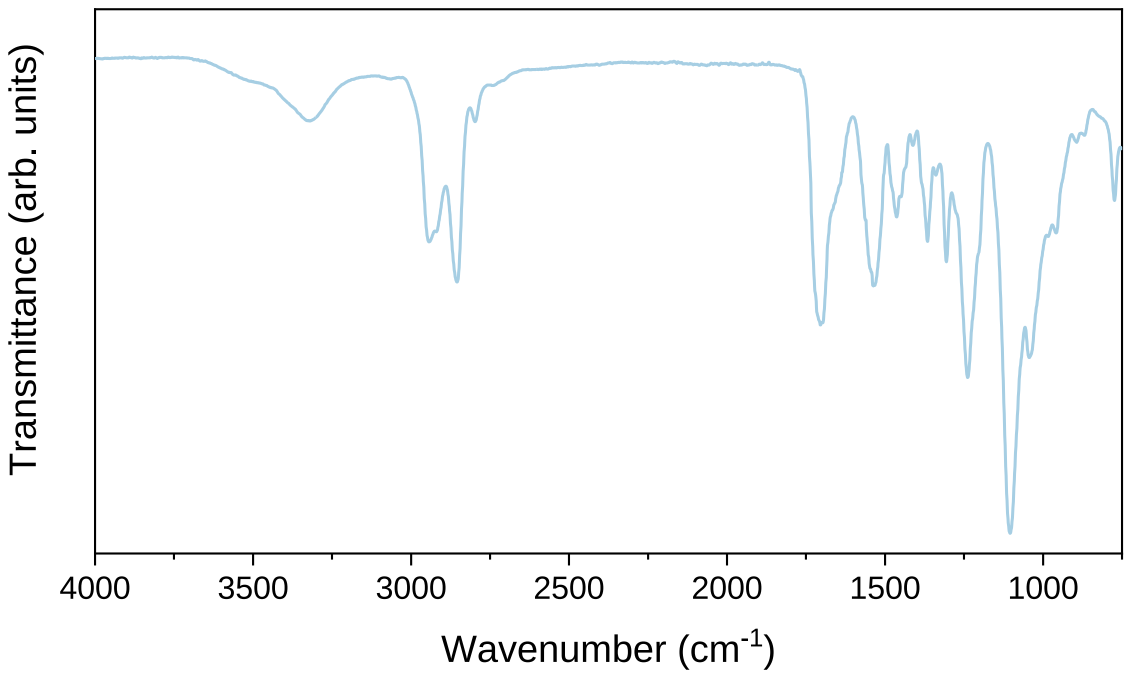


**Figure S2.** FTIR spectra of CPU from 4000 to 750 $cm^{-1}$. Absence of N=C=O stretching bands at 2265 $cm^{-1}$, indicating that excess isocyanate groups were removed from purification processes. Two absorption bands at approximately 3060-3750 $cm^{-1}$ and 1600-1800 $cm^{-1}$ corresponded to N-H and C=O stretching band respectively, revealing the formation of urethane (-NH-C=O-O-) groups. Lastly, peaks at 2800-3000 $cm^{-1}$ and 1104 $cm^{-1}$ were associated to the C-H stretching vibrations (asymmetric and symmetric aliphatic stretching modes) and C-O-C (ether oxygen of soft-segment stretching) respectively.

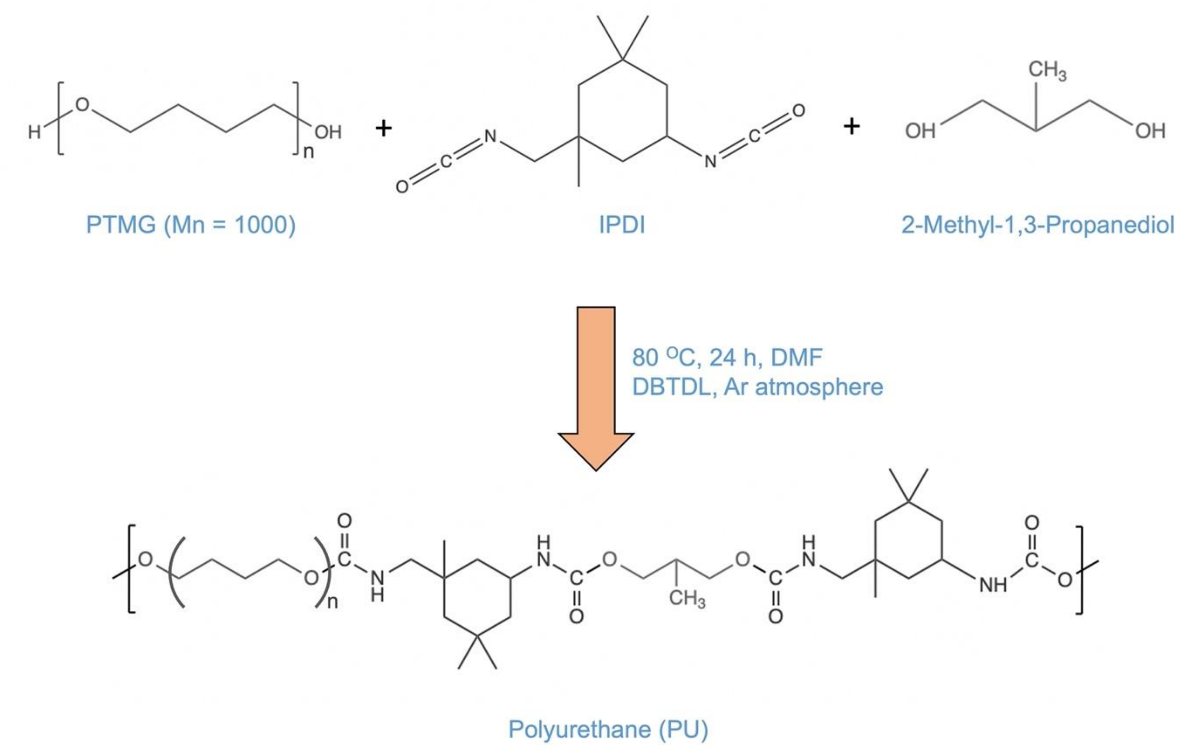


**Figure S3.** Synthesis route of control polyurethane.

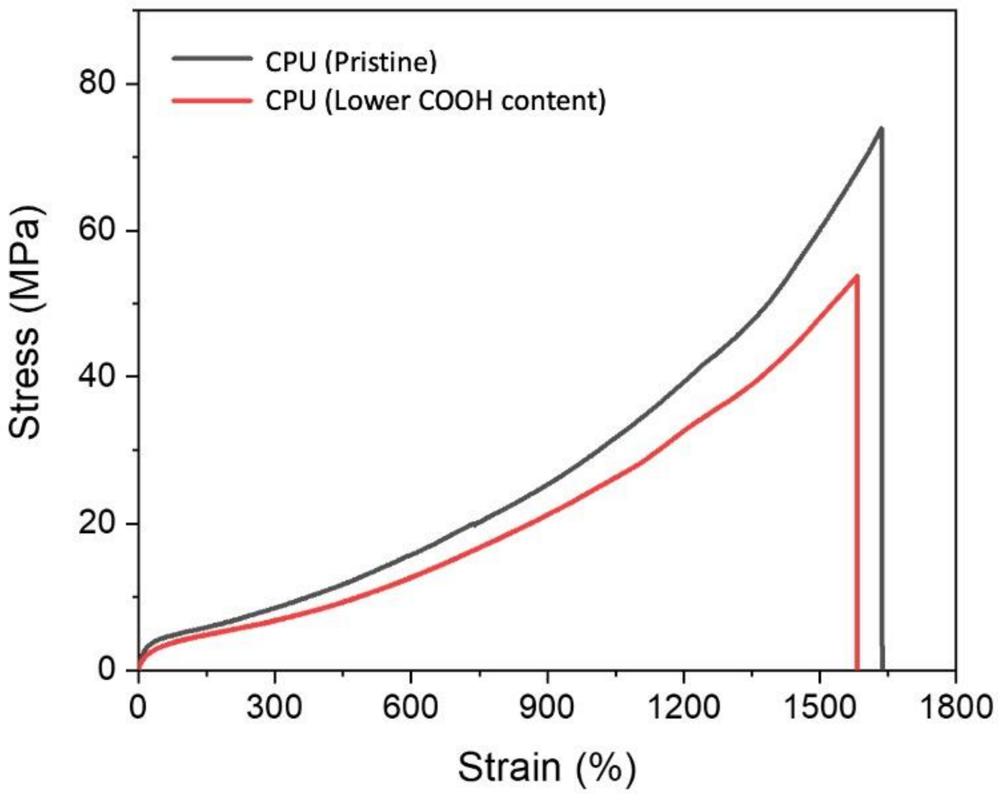


**Figure S4. Stress-strain curve of CPU with different carboxyl content.** 24 mmol of DMPA was utilized for pristine CPU that is studied in this work. 12 mmol of DMPA was utilized for CPU to show the impact of lower COOH content on the mechanical properties.

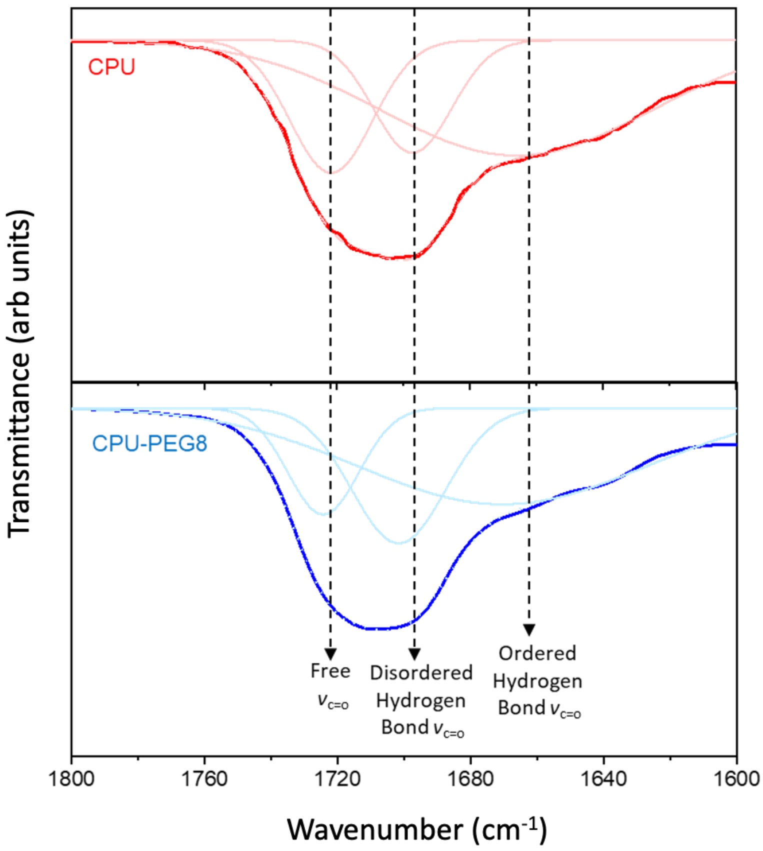


**Figure S5.** FTIR peak deconvolution of CPU and CPU-PEG8 at C=O stretching region (1800-1600 cm$^{-1}$). For CPU, peaks at 1721 cm$^{-1}$, 1697 cm$^{-1}$ and 1666 cm$^{-1}$ were assigned to free C=O, disordered hydrogen bonded C=O as well as ordered hydrogen bonded C=O. After the addition of PEG, a small shift was observed towards higher wavenumbers from 1666 cm$^{-1}$ to 1670 cm$^{-1}$ and 1697 cm$^{-1}$ to 1701 cm$^{-1}$ for disordered hydrogen bonded C=O and ordered hydrogen bonded C=O respectively. The shift towards higher wavenumbers indicates that the bond lengths were being reduced because of the disruptive effect of PEG plasticizers that minimized hydrogen bonding between carboxyl groups.[1, 2]

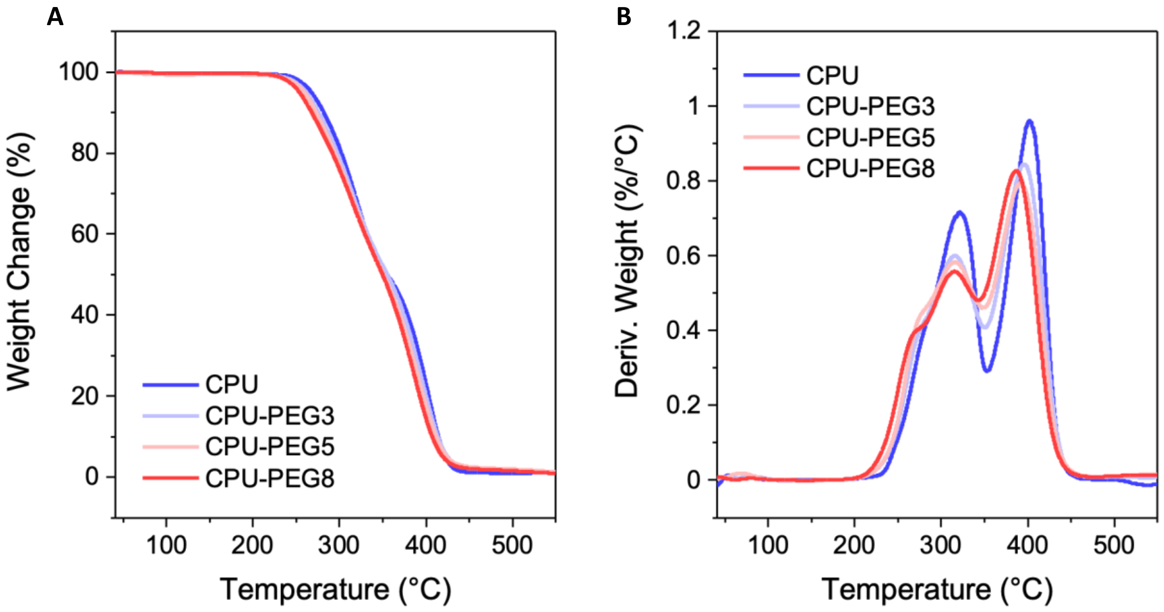


**Figure S6. Thermogravimetric analysis (TGA) of CPU and CPU-PEG blends (3, 5, 8 wt%). (A)** TGA spectrum and their **(B)** derivative from 40 to 550 $^{o}$C.

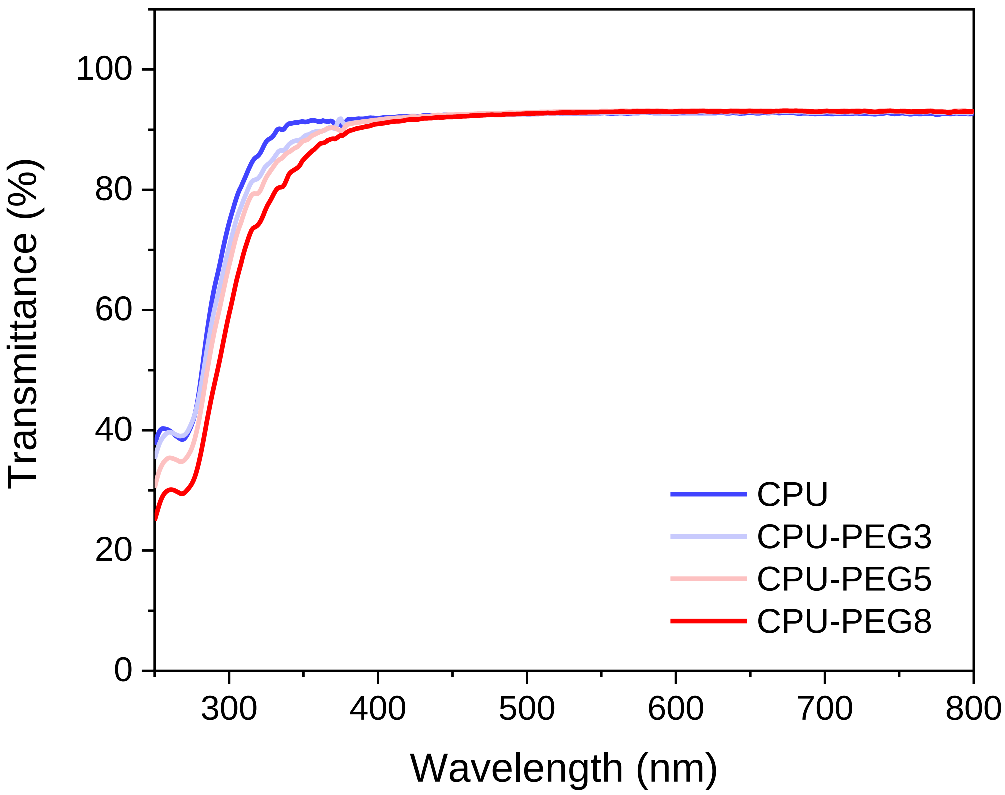


**Figure S7.** UV-Vis spectrum of CPU and CPU-PEG blends (3, 5 and 8 wt%) from 250 to 800 nm.

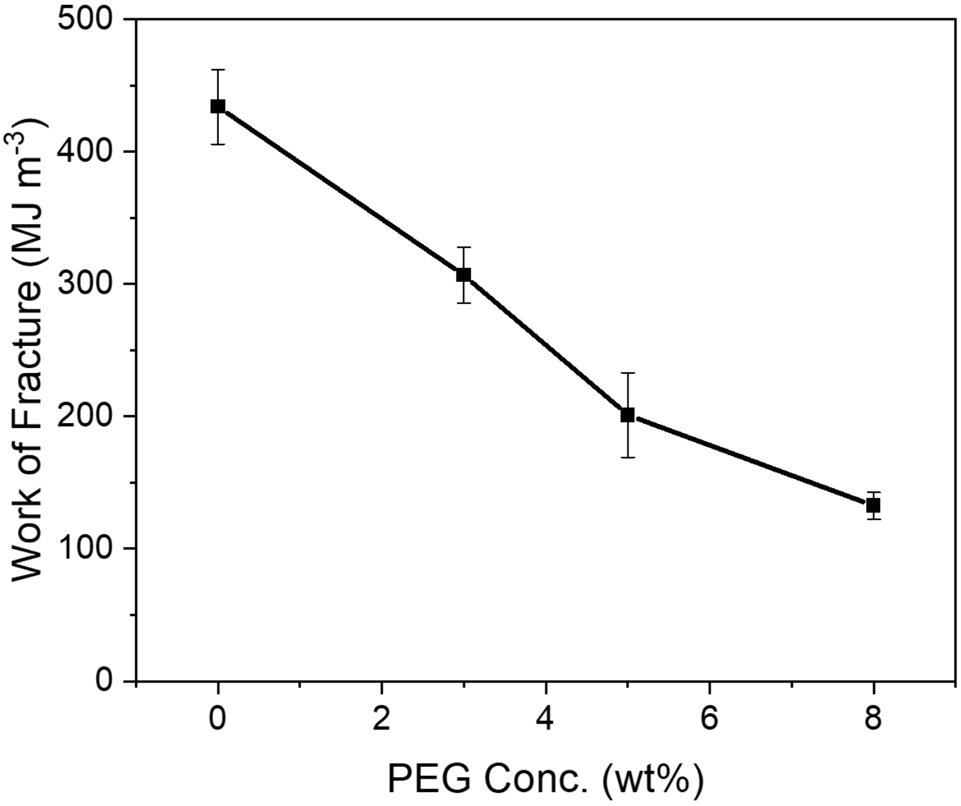


**Figure S8.** Work of fracture (integration of the stress-strain curve) for pristine CPU and CPU-PEG blends (3, 5 and 8 wt%). All error bars are the standard deviation of three independent samples.

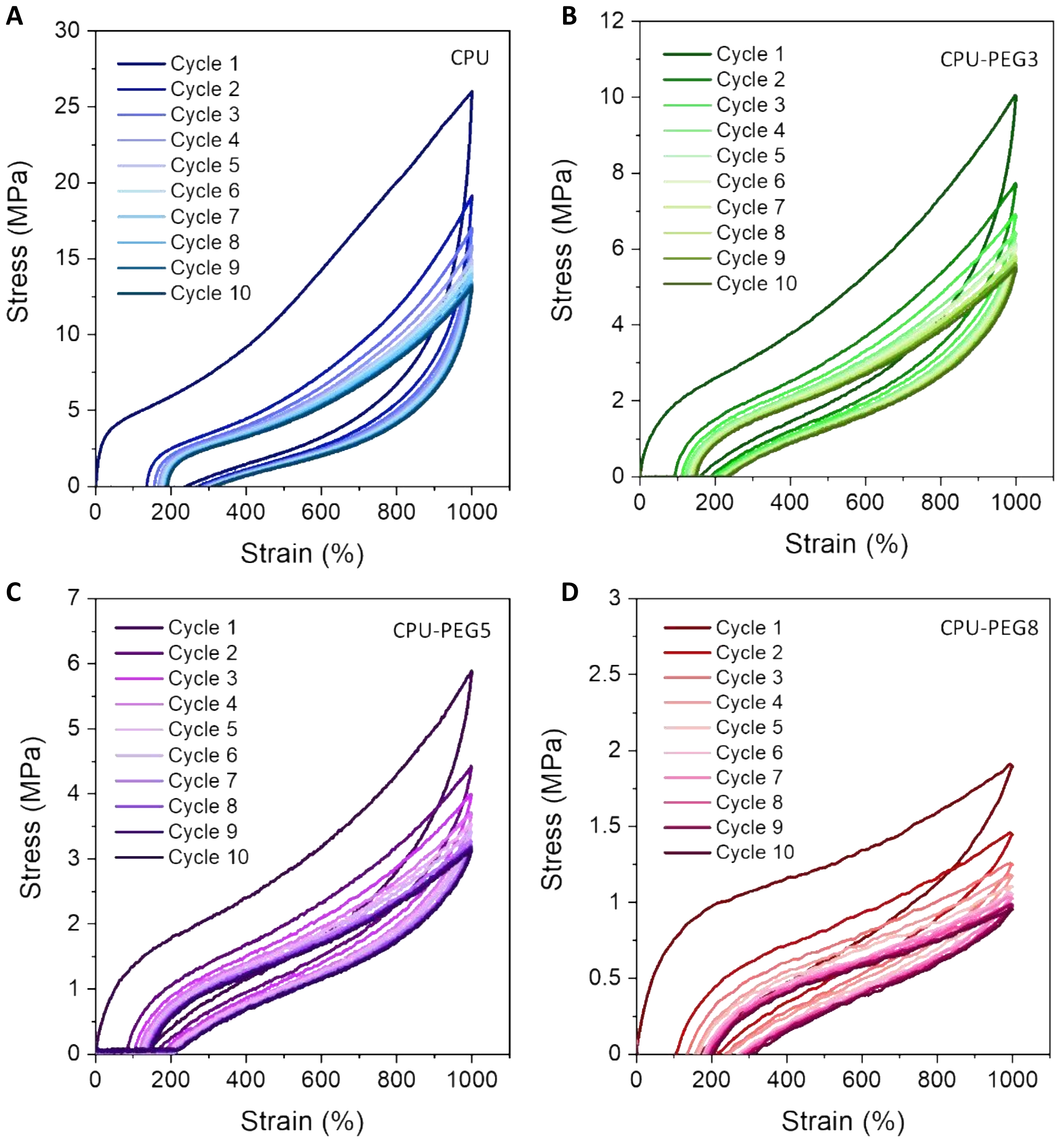


**Figure S9. Cyclic tensile tests.** Measurements on **(A)** CPU, **(B)** CPU-PEG3, **(C)** CPU-PEG5 and **(D)** CPU-PEG8 were performed at a strain rate of 100 mm $min^{-1}$ for 10 cycles.

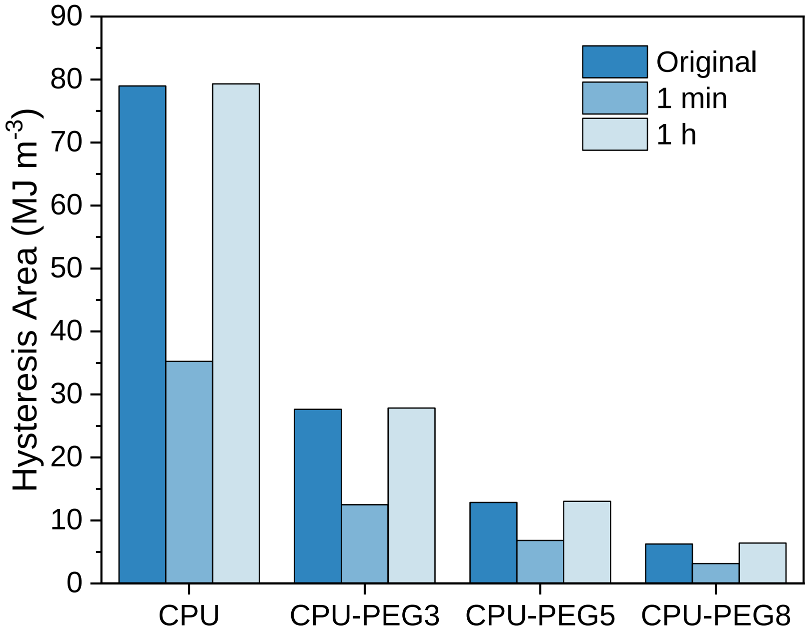


**Figure S10.** Hysteresis area of first cycle and after recovery for 1 min and 1 hour.

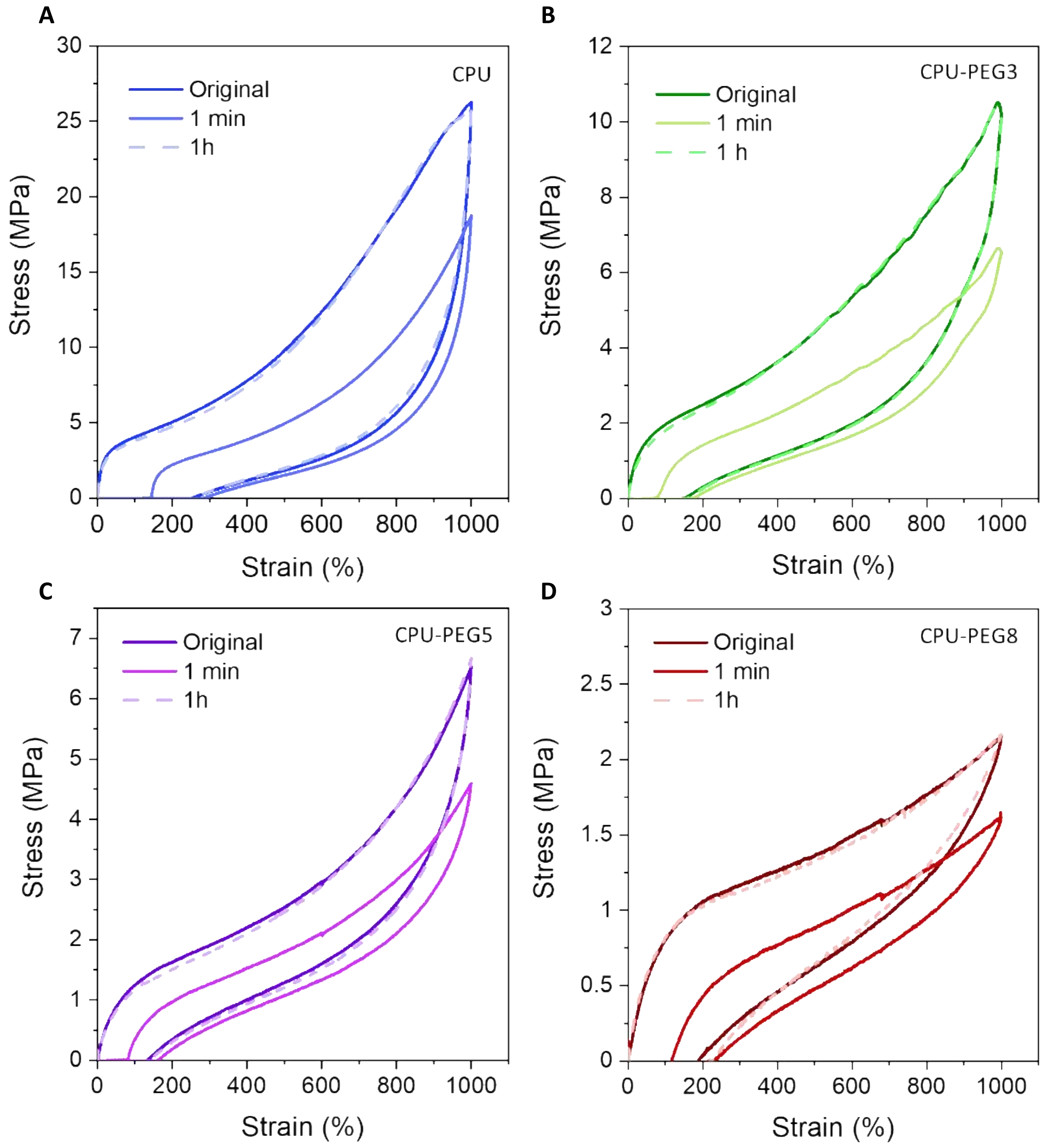


**Figure S11. Cyclic tensile tests after 1 min and 1 hour recovery.** Measurements on **(A)** CPU, **(B)** CPU-PEG3, **(C)** CPU-PEG5 and **(D)** CPU-PEG8 were performed at a strain rate of 100 mm $min^{-1}$.

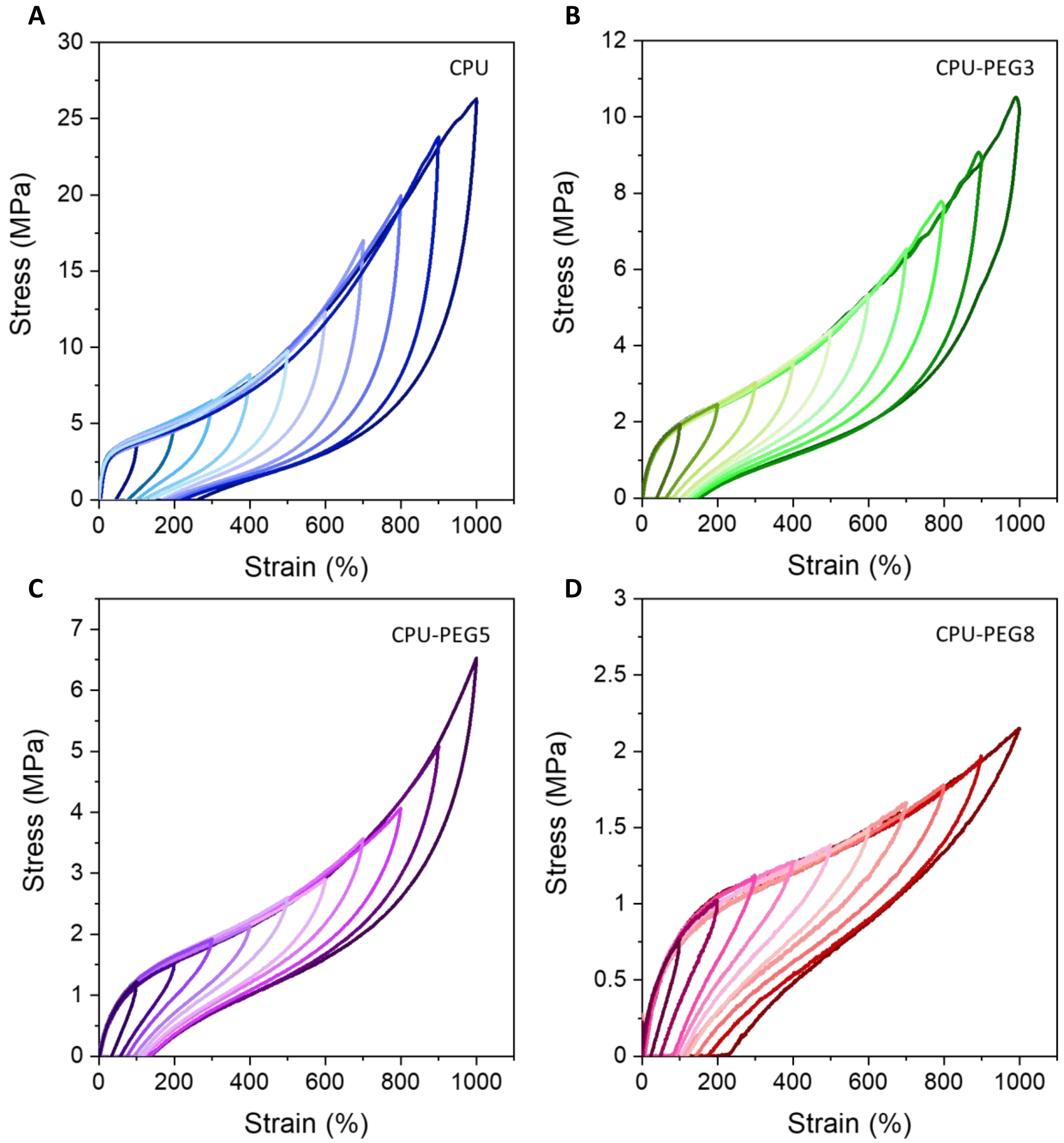


**Figure S12. Cyclic tensile test at different strain limits.** Measurements on **(A)** CPU, **(B)** CPU-PEG3, **(C)** CPU-PEG5 and **(D)** CPU-PEG8 were performed at a strain rate of 100 mm $min^{-1}$.

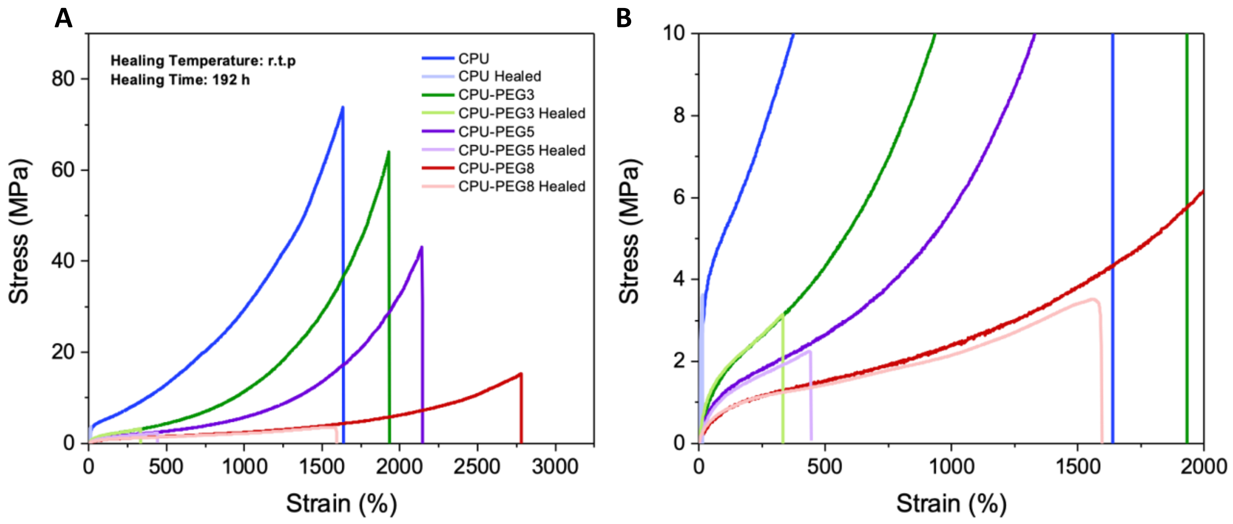


**Figure S13. Healing of mechanical properties at room temperature. (A)** Tensile tests of self-healed CPU and CPU-PEG blends after healing at room temperature for 192 h. (**B**) Magnified view at tensile stress from 0 to 10 MPa and tensile strain from 0 to 2000%.

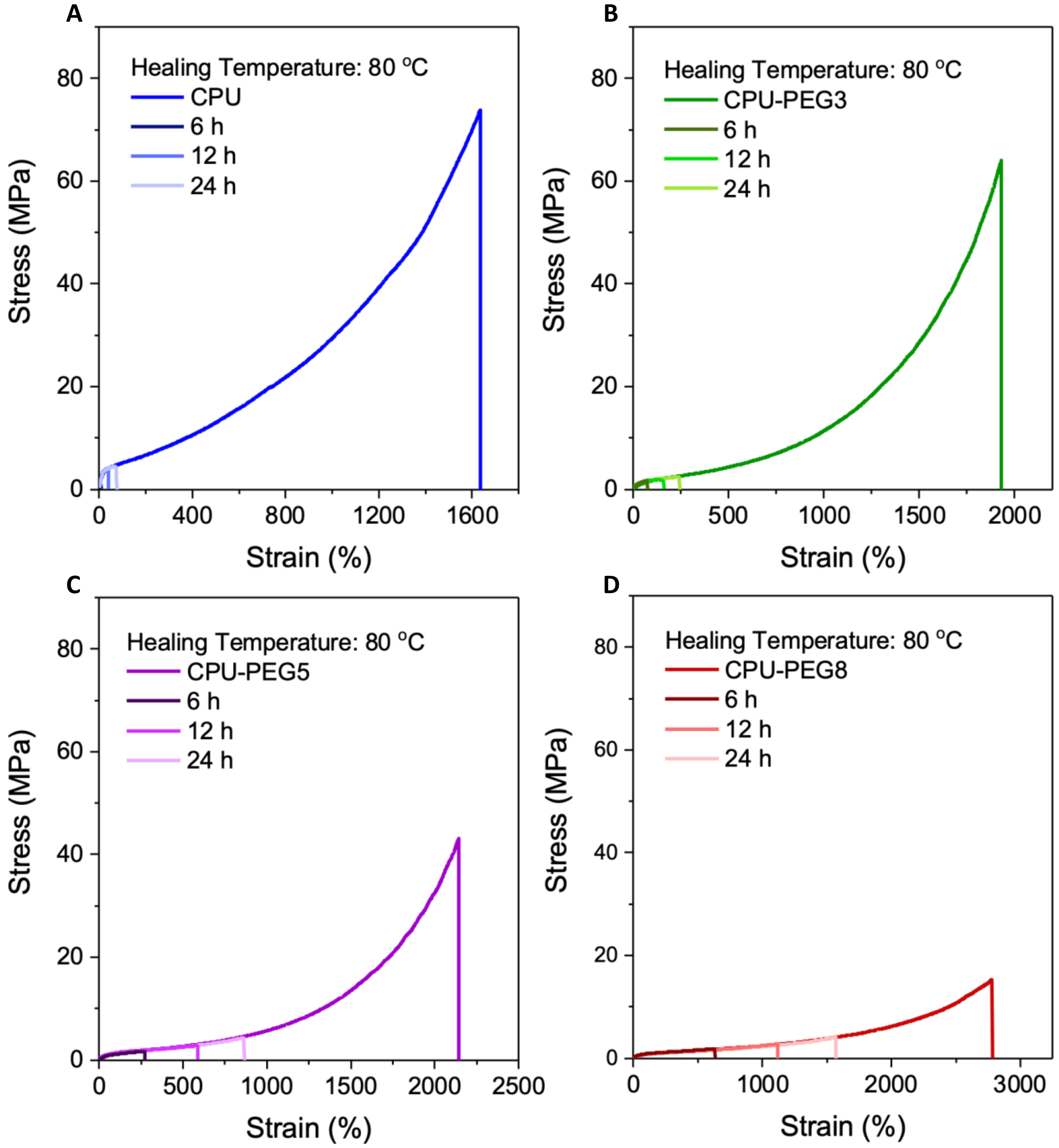


**Figure S14. Representative stress-strain curves before and after self-healing at 80 ºC . (A)** CPU, **(B)** CPU-PEG3, **(C)** CPU-PEG5 and **(D)** CPU-PEG8 were healed over different healing times (6, 12 and 24 h).

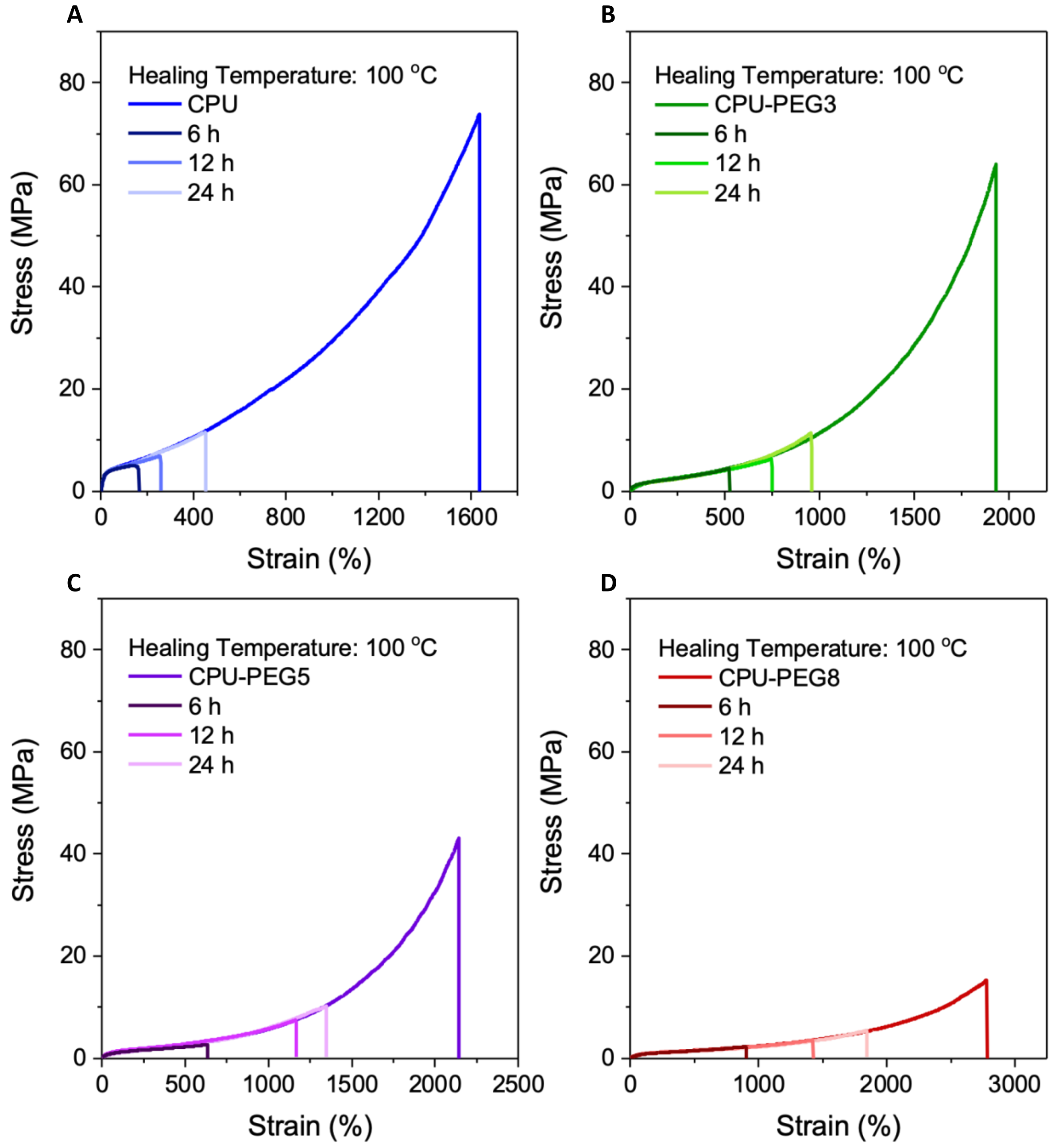


**Figure S15. Representative stress-strain curves before and after self-healing at 100 °C . (A)** CPU, **(B)** CPU-PEG3, **(C)** CPU-PEG5 and **(D)** CPU-PEG8 were healed over different healing times (6, 12 and 24 h).

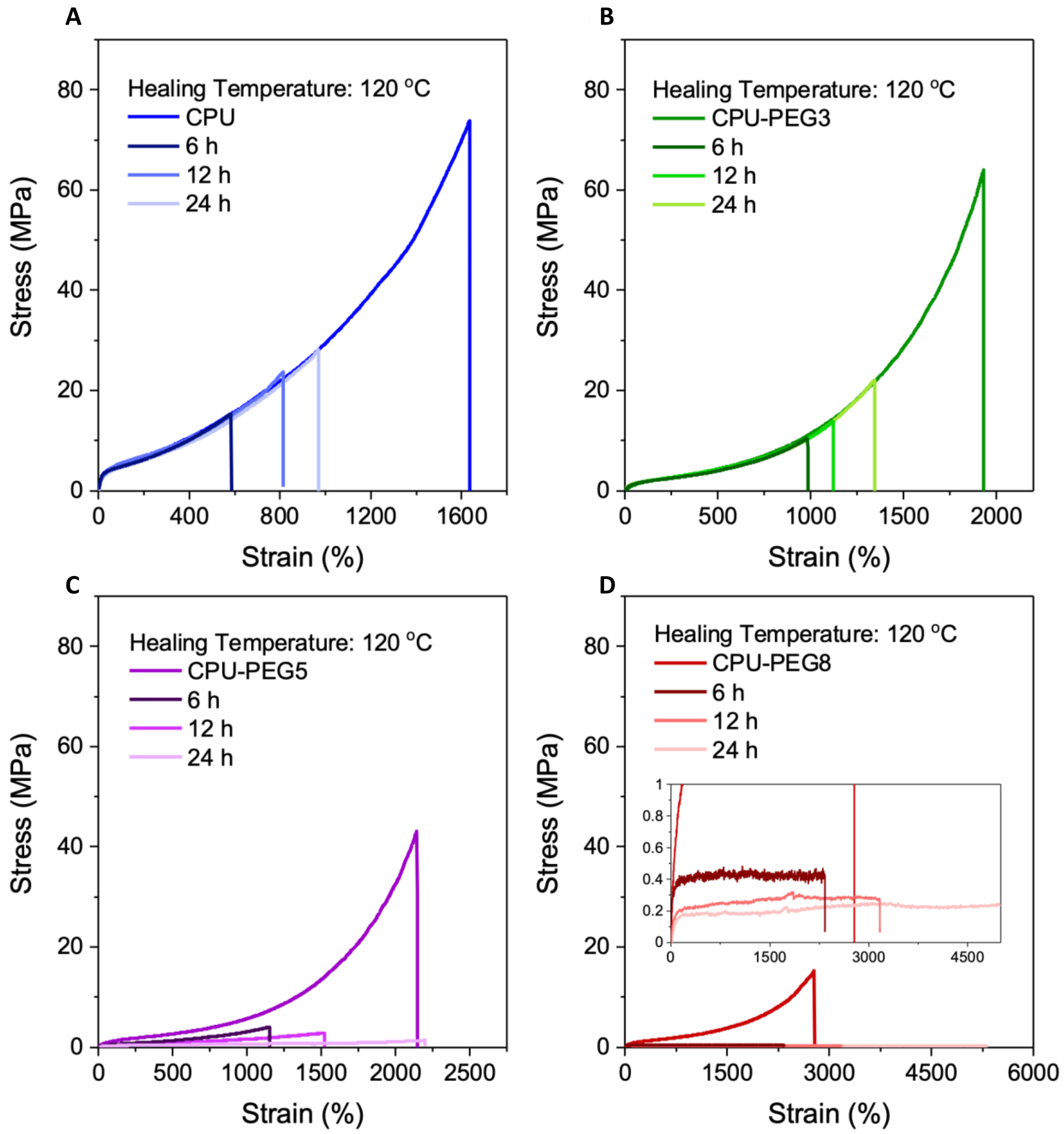


**Figure S16. Representative stress-strain curves before and after self-healing at 120 ºC. (A)** CPU, **(B)** CPU-PEG3, **(C)** CPU-PEG5 and **(D)** CPU-PEG8 (inset represents magnified view at lower tensile stress from 0 to 1 MPa) were healed over different healing times (6, 12 and 24 h).

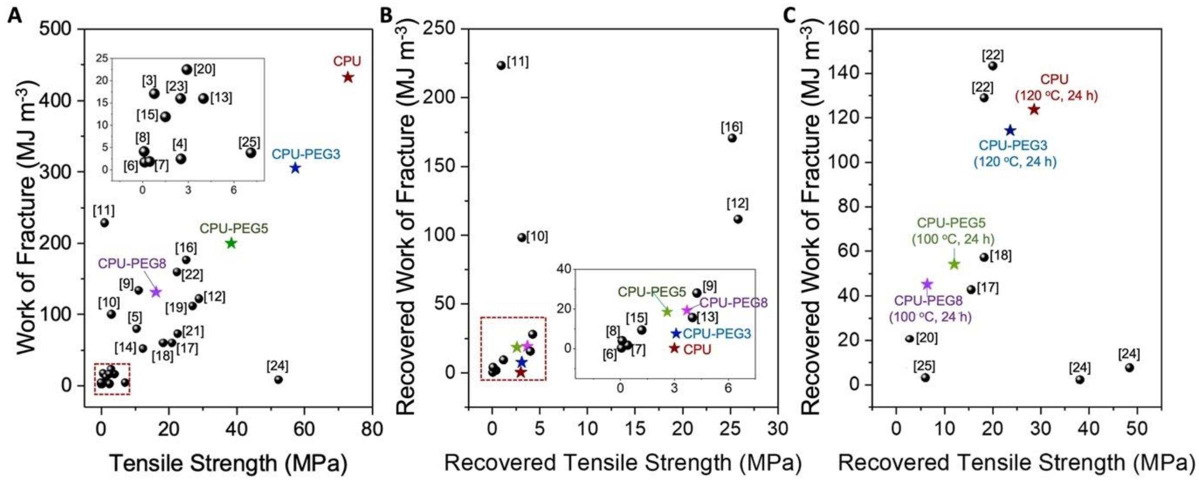


**Figure S17. Comparison with works on self-healing polymers.**[3-25] **(A)** Work of fracture and tensile strength of polymers before damage (inset represents magnified view indicated by the red dotted box). **(B)** Recovered work of fracture and tensile strength of polymers that self-healed in the range of room temperature to 60 $^{o}$C. (inset represents magnified view indicated by the red dotted box). **(C)** Recovered work of fracture and tensile strength of polymers that are self-healed at 80 $^{o}$C and above.

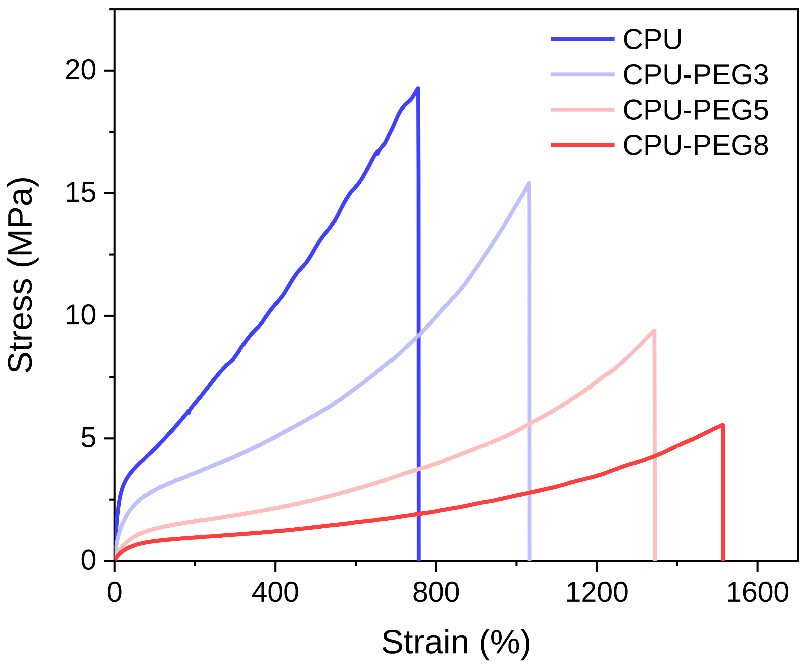


**Figure S18.** Representative stress-strain curves from pure shear tests performed on CPU and CPU-PEG blends.

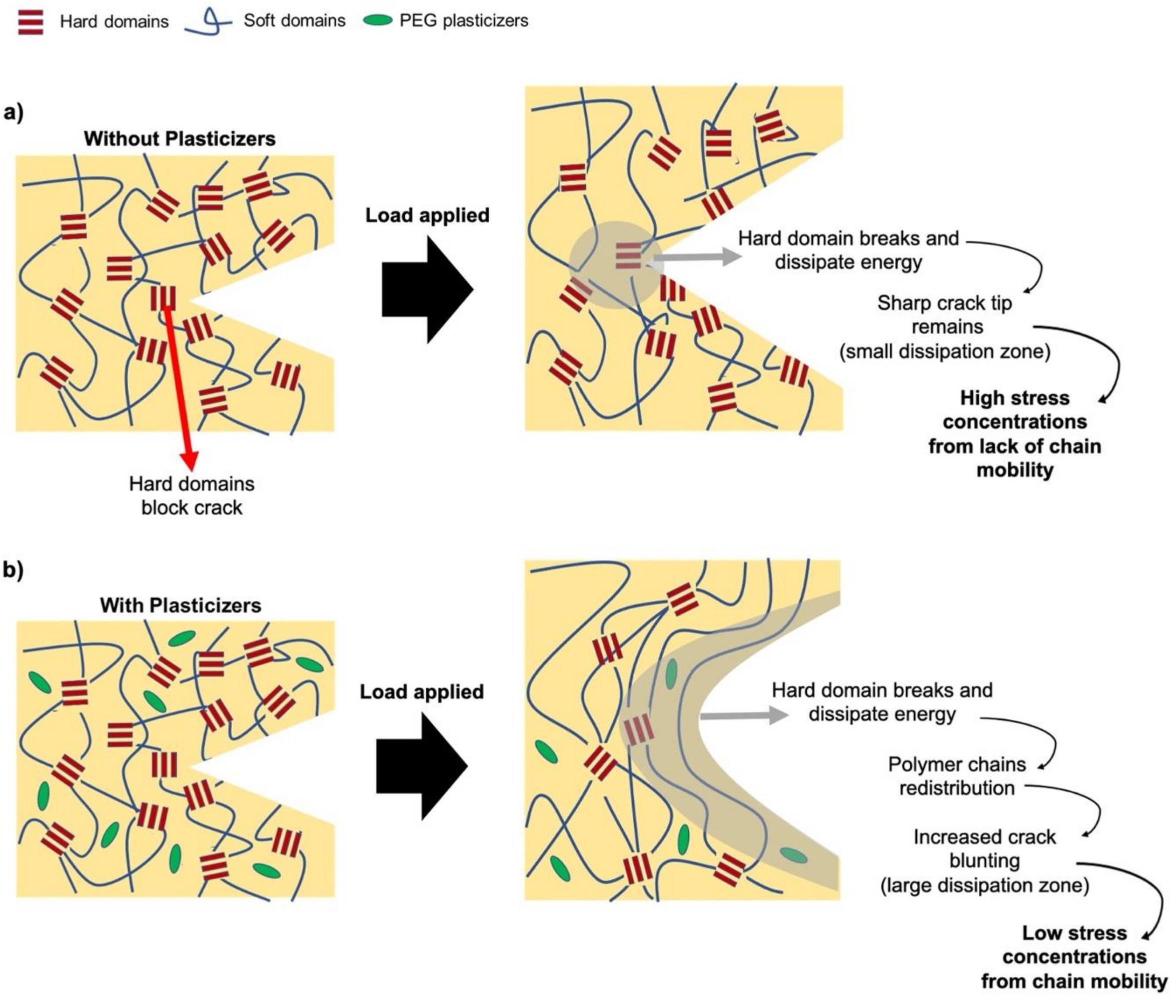


**Figure S19. Schematic of fracture toughening mechanism based on chain mobility. (A)** CPU with minimal chain mobility remains to have high stress concentrations at the crack tip. Improved fracture toughness of CPU is achieved by the large energy dissipated from the rupture of the strong hard domains. **(B)** CPU-PEG blends with improved chain mobility display crack blunting behaviors that allow stress concentrations to be dispersed at the crack tip after hard domains are ruptured. This provides greater notch insensitivity with crack initiation occurring at higher strains. By optimizing the amount of chain mobility and strong hard domains, enhanced fracture toughness is achieved.

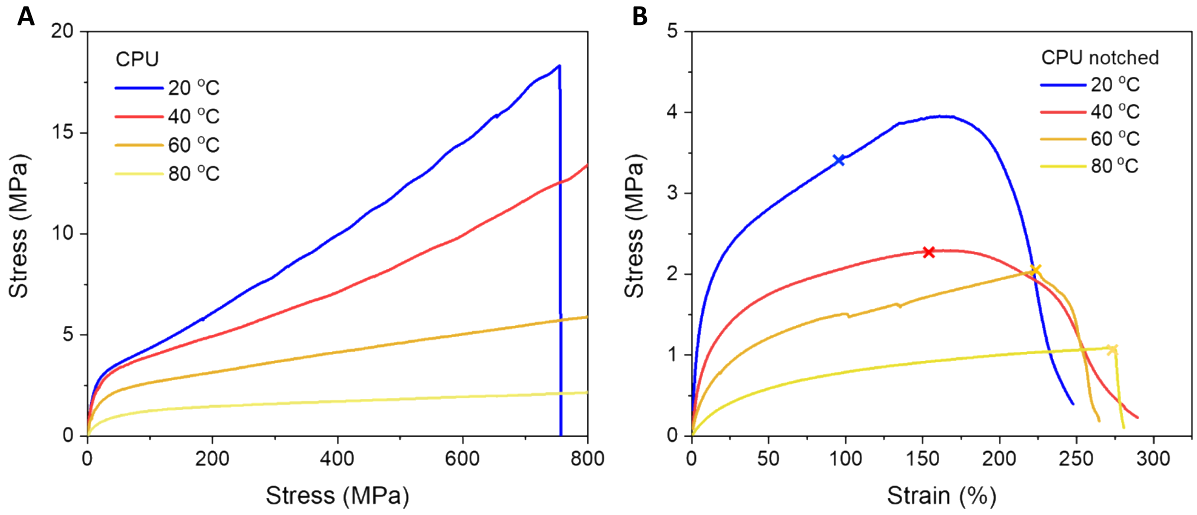


**Figure S20. Pure shear tests at different temperatures.** Representative stress-strain curves of **(A)** CPU and **(B)** notched CPU. The cross indicates the point where crack advancement was initiated, and the corresponding strain represents the critical strain.

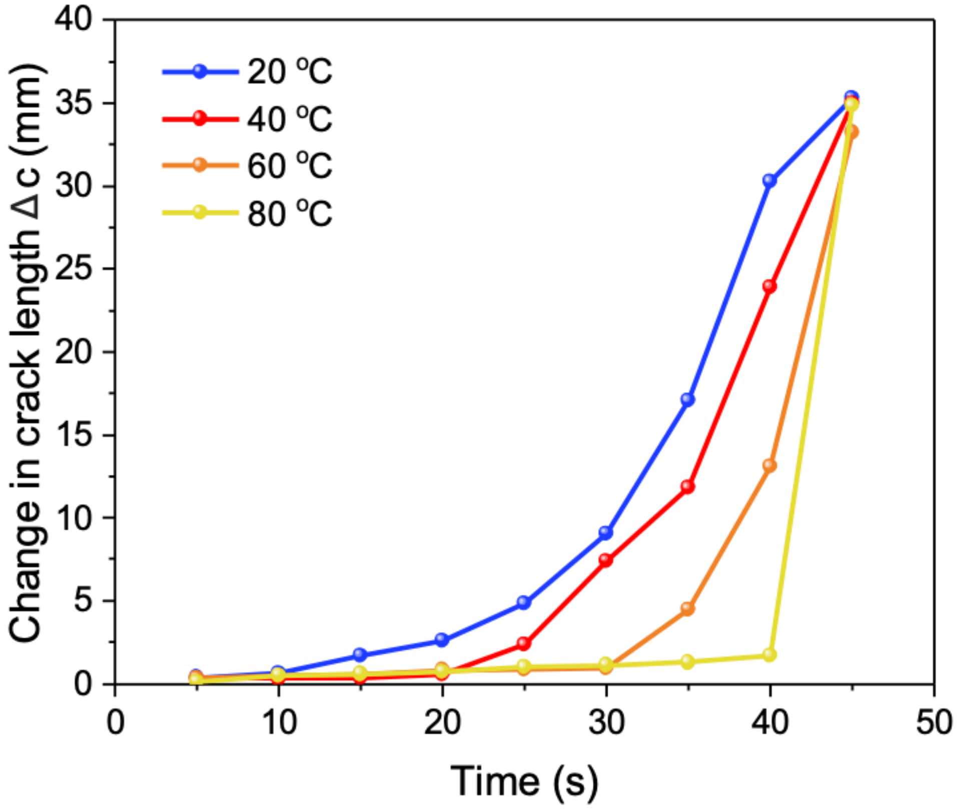


**Figure S21.** Change in crack length within CPU during pure shear test at different temperatures.

**Table S1 Molecular weight of CPU and control PU**

| Material | $M_n$ (g mol$^{-1}$) | $M_w$ (g mol$^{-1}$) | PDI |
|---|---|---|---|
| CPU | 6641 | 10216 | 1.54 |
| Control PU | 6461 | 11786 | 1.82 |

**Table S2: Mechanical properties of CPU and CPU-PEG blends**

| Material | $Y_{10\%}$ (MPa)[a] | $Y_{100\%}$ (MPa)[b] | $Y_{200\%}$ (MPa)[b] | Tensile Strength (MPa) | Tensile Strain (%) | Work of Fracture (MJ m$^{-3}$) |
|---|---|---|---|---|---|---|
| CPU | 16.69 ± 4.81 | 1.49 ± 0.21 | 1.62 ± 0.13 | 72.6 ± 5.4 | 1675.5 ± 56.7 | 433.9 ± 28.3 |
| CPU-PEG3 | 4.56 ± 0.43 | 0.85 ± 0.04 | 0.68 ± 0.10 | 57.0 ± 6.2 | 1914.9 ± 30.3 | 306.7 ± 21.2 |
| CPU-PEG5 | 2.66 ± 0.60 | 0.57 ± 0.06 | 0.36 ± 0.09 | 38.1 ± 6.8 | 2033.9 ± 96.3 | 201.0 ± 32.0 |
| CPU-PEG8 | 1.58 ± 0.28 | 0.32 ± 0.10 | 0.20 ± 0.12 | 15.9 ± 1.3 | 2788.2 ± 50.2 | 132.6 ± 10.2 |

Standard deviations are based on three independent samples [a] Elastic modulus at 10% strain. Based on the slope of the stress-strain curves with a linear fit in the first 10% strain. [b] The elastic modulus at the respective strain is determined from the slope of the stress-strain curve using a linear fit within ± 10% strain.

**Table S3: Healing efficiency of CPU and CPU-PEG blends after 24 h at 80, 100 and 120 °C**

| Material | Healing Temperature (°C) | Healing Efficiency, $Y_{10\%}$ (%) | Healing Efficiency, Tensile Strength (%) | Healing Efficiency, Tensile Strain (%) | Healing Efficiency, Work of Fracture (%) |
|---|---|---|---|---|---|
| CPU | 80 | 129.0 ± 20.0 | 6.3 ± 1.2 | 4.4 ± 0.1 | 0.7 ± 0.1 |
| | 100 | 138.8 ± 16.0 | 16.3 ± 0.7 | 27.5 ± 0.3 | 7.3 ± 0.2 |
| | 120 | 129.2 ± 15.4 | 39.4 ± 3.6 | 58.1 ± 1.5 | 28.5 ± 2.8 |
| CPU-PEG3 | 80 | 131.3 ± 16.6 | 5.7 ± 0.5 | 13.3 ± 0.8 | 1.6 ± 0.2 |
| | 100 | 103.1 ± 15.3 | 21.7 ± 1.4 | 51.9 ± 2.1 | 15.2 ± 1.0 |
| | 120 | 117.5 ± 9.8 | 41.5 ± 3.3 | 72.9 ± 2.6 | 37.3 ± 4.5 |
| CPU-PEG5 | 80 | 105.3 ± 7.7 | 12.0 ± 2.7 | 42.0 ± 1.8 | 10.3 ± 1.2 |
| | 100 | 96.0 ± 11.0 | 31.5 ± 3.3 | 67.3 ± 1.3 | 27.0 ± 1.2 |
| | 120 | 22.1 ± 6.4 | 3.3 ± 0.4 | 104.3 ± 12.2 | 6.7 ± 1.5 |
| CPU-PEG8 | 80 | 103.2 ± 33.6 | 28.7 ± 3.1 | 59.9 ± 3.5 | 25.5 ± 2.0 |
| | 100 | 125.3 ± 27.5 | 40.0 ± 4.4 | 67.5 ± 3.9 | 34.1 ± 4.2 |
| | 120 | 21.5 ± 12.2 | 2.1 ± 0.6 | 191.2 ± 0.03 | 10.4 ± 1.8 |

Standard deviations are based on three independent samples. Healing efficiencies are derived from the property ratio between healed and pristine samples.

## References

[1] S. Park, G. Thangavel, K. Parida, S. Li, P. S. Lee, Advanced Materials **2019**, 31, 1805536.
[2] D. Chattopadhyay, A. J. Muehlberg, D. C. Webster, Progress in Organic Coatings **2008**, 63, 405.
[3] P. Shi, Y. Wang, K. Wan, C. Zhang, T. Liu, Advanced Functional Materials **2022**, 32, 2112293.
[4] J. Yang, G. Zhang, X. Zhou, L. Xiao, G. Hao, J. Luo, H. Zong, Y. Wang, W. Jiang, ACS Applied Polymer Materials **2023**, 5, 3005.
[5] J. Chen, Y. Gao, L. Shi, W. Yu, Z. Sun, Y. Zhou, S. Liu, H. Mao, D. Zhang, T. Lu, Nature Communications **2022**, 13, 4868.
[6] Z. Zhang, N. Ghezawi, B. Li, S. Ge, S. Zhao, T. Saito, D. Hun, P. F. Cao, Advanced Functional Materials **2021**, 31, 2006298.
[7] Y. Yang, F.-S. Du, Z.-C. Li, ACS Applied Polymer Materials **2020**, 2, 5630.
[8] D.-P. Wang, Z.-H. Zhao, C.-H. Li, ACS Applied Materials & Interfaces **2021**, 13, 31129.
[9] D. Zhao, X. Zhou, Q. Li, J. Yang, H. Li, Materials Horizons **2022**, 9, 2626.
[10] D. Wang, D. Liu, J. Xu, J. Fu, K. Wu, Materials Horizons **2022**, 9, 640.
[11] M. Li, L. Chen, Y. Li, X. Dai, Z. Jin, Y. Zhang, W. Feng, L.-T. Yan, Y. Cao, C. Wang, Nature Communications **2022**, 13, 2279.
[12] Y. Li, W. Li, A. Sun, M. Jing, X. Liu, L. Wei, K. Wu, Q. Fu, Materials Horizons **2021**, 8, 267.
[13] W. Wang, W. Wang, F. Wang, X. Xie, G. Yi, Z. Li, Journal of Materials Chemistry A **2022**, 10, 23375.
[14] W. Yang, Y. Zhu, T. Liu, D. Puglia, J. M. Kenny, P. Xu, R. Zhang, P. Ma, Advanced Functional Materials **2023**, 33, 2213294.
[15] F. Kang, Y. Yang, W. Wang, Z. Li, European Polymer Journal **2023**, 184, 111794.
[16] W. B. Ying, G. Wang, Z. Kong, C. K. Yao, Y. Wang, H. Hu, F. Li, C. Chen, Y. Tian, J. Zhang, Advanced Functional Materials **2021**, 31, 2009869.
[17] J. Liu, J. Liu, S. Wang, J. Huang, S. Wu, Z. Tang, B. Guo, L. Zhang, Journal of Materials Chemistry A **2017**, 5, 25660.
[18] D. Liu, C.-J. Fan, Y. Xiao, K.-K. Yang, Y.-Z. Wang, Polymer **2022**, 263, 125513.
[19] H. Xie, D. Sheng, Y. Zhou, S. Xu, H. Wu, X. Tian, Y. Sun, X. Liu, Y. Yang, New Journal of Chemistry **2020**, 44, 13584.
[20] X. Zhou, Z. Gong, J. Fan, Y. Chen, Polymer **2021**, 237, 124357.
[21] X. Wu, M. Liu, J. Zhong, Y. Zhong, J. Rong, F. Gao, Y. Qiao, L. Shen, H. He, New Journal of Chemistry **2022**, 46, 13415.
[22] H. Chen, Z. Sun, H. Lin, C. He, D. Mao, Advanced Functional Materials **2022**, 32, 2204263.
[23] L. Bai, P. Qv, J. Zheng, Journal of Materials Science **2020**, 55, 14045.
[24] Z. Luo, B. Yang, F. Liu, X. Pan, Y. Zeng, ACS Applied Polymer Materials **2023**, 5, 8670.
[25] L. Bai, J. Zheng, Composites Science and Technology **2020**, 190, 108062.
